# Membrane lipid remodeling under salt stress is associated with salt tolerance in lettuce

**DOI:** 10.1186/s12870-026-09169-3

**Published:** 2026-06-20

**Authors:** Modan K. Das, Neil D. Adhikari, Ivan Simko, Ainong Shi, Beiquan Mou

**Affiliations:** 1 U.S. Department of Agriculture-Agricultural Research Service, Sam Farr United States Crop Improvement and Protection Research Center, Salinas, CA 93905 USA; 2 Department of Horticulture, University of Arkansas, Fayetteville, AR 72701 USA; 3 Present Address: California Department of Public Health, 1616 Capitol Ave, Sacramento, CA 95814 USA

**Keywords:** Lettuce, *Lactuca sativa L*., Membrane lipid, Salt tolerance, Lipid remodeling under salt stress

## Abstract

**Background:**

Salt stress is one of the most important abiotic stresses limiting crop production in the salinity affected growing regions. Lettuce (*Lactuca sativa* L.) is sensitive to salinity. Understanding the physiological and biochemical mechanisms involved in salt tolerance would facilitate developing strategies to breed lettuce cultivars with improved salt tolerance. Membrane lipid remodeling has been shown to play important roles in adaptation and tolerance to salt stress in several crop species; however, such information is lacking for lettuce. Accordingly, we conducted a controlled growth chamber experiment to investigate membrane lipid remodeling under salt stress using four pairs of lettuce genotypes - each pair consisting of a salt-tolerant and a salt-sensitive genotype from the same horticultural type.

**Results:**

Based on the 15 lipid classes analyzed, the lettuce leaf lipidome consisted of 79% phospholipids, 20% galactolipids and ~ 1% triacylglycerols (TAGs). Phosphatidylcholine (PC) and phosphatidylethanolamine (PE) were the predominant phospholipids accounting for 36% and 34% of the total phospholipids, respectively. Under salt stress, both content and unsaturation levels of galactolipids and TAGs increased, whereas content and unsaturation levels of phospholipids, except phosphatidylglycerol (PG) decreased. DGDG (digalactosyldiacylglycerol):MGDG (monogalactosyldiacylglycerol) ratio remained stable under salt stress, whereas PC:PE ratio increased. The changes in the PC:PE ratio had a significant correlation with changes in fresh weight (FW) indicating that PC: PE ratio had a role in salt tolerance. Correlation and regression analyses indicated significant relationships of changes in FW with changes in the content of the lipids PG, PC, phosphatidic acid (PA), TAG 16:0, TAG 18:2, TAG 18:1, and TAG 18:0 due to salt stress, suggesting that these lipid classes were involved in salt tolerance in lettuce in the present study.

**Conclusions:**

This study demonstrates that lettuce undergoes significant membrane lipid remodeling under salt stress, and these changes contribute to salt tolerance. Using lipid profiles from control plants and salt-induced reductions in FW, we developed regression models which could potentially be used to predict FW losses due to salt stress based on MGDG and DGDG content in lettuce grown under control or normal conditions. These findings highlight key lipid traits that may be used in breeding or screening strategies to improve salt tolerance in lettuce.

**Supplementary Information:**

The online version contains supplementary material available at https://doi.org/10.1186/s12870-026-09169-3.

## Background

Throughout their lifecycle, plants are exposed to different biotic and abiotic stresses. Salt stress is one of the most important abiotic stresses for plants growing in salinity affected growing regions. Recently, the Food and Agriculture Organization (FAO) reported that globally more than 424 million hectares of topsoil and 833 million hectares of subsoil are salt-affected (https://www.fao.org/soils-portal/data-hub/soil-maps-and-databases/global-map-of-salt-affected-soils/en/). As a result, many farmers do not have much option but to grow crops in salt-affected soils. Therefore, developing salt tolerant crop cultivars for this salinity affected regions is essential.

Lettuce (*Lactuca sativa* L.) is an autogamous annual crop species with a diploid chromosome number of 2n = 18. Commercially, lettuce is one of the most important leafy vegetable crops globally. Based on leaf size, shape and texture, head formation and stem type, the cultivated lettuce is generally classified into six horticultural types namely crisphead, butterhead, romaine, leaf, stem, and Latin [1]. In 2022, the U.S. growers earned $1.54 billion, $1.33 billion, and $1.25 billion, respectively, from the sales of romaine, iceberg (crisphead), and leaf types of lettuce (https://www.ers.usda.gov/data-products/chart-gallery/gallery/chart-detail/?chartId=106516). More than 70% of the U.S. lettuce is grown in California, with the main producing region located in the Salinas Valley [2] and the soil salinity in the Salinas Valley is becoming a growing concern especially due to its proximity to the ocean [3]. Environmental changes have contributed to higher sea levels and farther saltwater intrusion. Rising temperatures promote water transpiration from plants and evaporation from soil, leaving more salts behind in land. Seeds of lettuce are planted in the topsoil where salt levels are often the highest. Lettuce is sensitive to salinity and baby lettuce crop is especially vulnerable to salt stress.

Substantial variation in salt tolerance among 178 lettuce cultivars and germplasm accessions from four major horticultural types (butterhead, crisphead, romaine, and leaf) and wild type has been reported [4]. Based on the existence of salt tolerant plant species (halophytes) and differences in salt tolerance between genotypes within salt sensitive plant species (glycophytes), it was suggested that there is a genetic basis to salt tolerance [5]. Research efforts have been focused on identifying genetic diversity and investigating the genetic basis of salt tolerance and their use in developing salt tolerant crop cultivars of different plant species including lettuce [6–14]. Understanding the physiological and biochemical mechanisms involved in plant adaptation to salt stress is essential for fully exploring the genetic basis of salt tolerance. Therefore, many research efforts have also been directed toward unraveling these mechanisms of salt stress adaptation [15–21]. However, reports of such research in lettuce are limited [22, 23].

Being the interface between the cell and environment, plant cell membranes are the sites of sensing and initiating rapid responses to changes in environmental conditions including salt stress [18, 24]. The cell membrane is composed of about 50% lipids and 50% proteins [25]. Lipids provide the basic structural organization of membranes, while membrane proteins carry out the specific functions of the different membranes of the cell. In addition to providing the basic structure, membrane lipids also play key roles in regulating the membrane integrity, fluidity, permeability, protein transport, activities of membrane-bound enzymes, and lipid signaling [26–30]. The three main classes of lipids in plant cell membranes are: glycerolipids, sphingolipids, and sterols [31]. Of these, the most abundant class is the glycerolipids, which is further divided into four types, namely phospholipids, galactolipids, sulfolipids, and TAGs, with phospholipids being the major one [31–33]. The phospholipids in the cell membrane form lipid bilayer, which provides a stable barrier between two aqueous compartments and represents the basic structure of all biological membranes [34]. In recent years, phospholipids have been a key focus of research due to their important role in regulating cellular activities such as membrane rearrangement, cytoskeletal dynamics, phosphorus deficiency response, cold stress response, and salt stress response [28, 35–39].

The galactolipids MGDG and DGDG and the sulfolipid sulfoquinovosyldiacylglycerol (SQDG) exist in the thylakoid membranes. MGDG (~ 50%), DGDG (~ 25–30%), SQDG (~ 10–15%), and the phospholipid PG (~ 10–15%) establish the thylakoid membranes and are integral constituents of the photosynthetic complexes [40]. Only DGDG, SQDG, and PG, which are cylindrically shaped lipids, are capable of spontaneously assembling into bilayer structures. On the other hand, MGDG, due to its conical shape, preferentially forms non-bilayer phases, such as inverted hexagonal, isotropic and cubic phases [41–44]. Also, lipid mixtures containing more than about 30% MGDG no longer form lipid vesicles [45]. In plants, TAGs are stored mainly as a high-energy storage lipid within lipid droplets (LDs) in seeds or fruits [46, 47]. Typically, TAGs are not produced at significant levels in vegetative tissues, however, a small amount of TAGs are present in these tissues, where they play a role in the sequestration of toxic lipid intermediates such as diacylglycerol (DAG) and free fatty acids following abiotic stress [46, 48, 49].

The primary effects of salt stress on cells are osmotic and ionic or ion-toxicity effects, while the secondary effects of salt stress are complex including oxidative stress; damage to cellular components such as membrane lipids, proteins and nucleic acids; and metabolic dysfunction [50]. Plants adapt to these adverse effects by remodeling membrane lipids and by doing this, plants maintain membrane structural integrity, permeability, and functionality [51]. Membrane lipid remodeling under salt stress involves changes in lipid content, proportion, and composition [52].

Changes in membrane lipid composition includes modification of lipid head groups and/or their acyl chain length and increase in fatty acid unsaturation, since the ‘kinks’ in the unsaturated fatty acid structure promote membrane fluidity [52–55]. Guo et al. [56] published a review on membrane lipid remodeling in plants under salt stress and presented a model depicting interaction of membrane lipid with membrane protein in imparting salt tolerance. Remodeling of membrane lipids in response to salt stress have been reported in several diverse plant species including rice, wheat, barley, soybean, and sweet potato [16, 18–21, 53, 57–62]. However, to our knowledge, such information is lacking in lettuce. Accordingly, the objectives of the present study were to investigate membrane lipid remodeling in response to salt stress and its role in salt tolerance using four salt-tolerant and four salt-sensitive lettuce genotypes.

## Methods

### Plant materials

Based on earlier ratings of salinity tolerance [4], eight lettuce cultivars and germplasm accessions (hereafter referred to as ‘genotype’) were selected for the current study. All eight genotypes were obtained from the lettuce germplasm collection maintained at the United States Department of Agriculture - Agricultural Research Service, Salinas, CA. Genotypes from the same/similar horticultural type were compared to each other to minimize the effect of horticultural types on observed differences in the measured traits/parameters. The selected genotypes were: ‘Laura’ (crisphead type, tolerant to salinity, abbreviated in tables and figures as LAU), ‘Early Bird’ (crisphead, sensitive to salinity, EAB), ‘Morgana’ (butterhead, tolerant, MOR), ‘Mayfair’ (butterhead, sensitive, MAY), ‘Eruption’ (Latin, tolerant, ERU), ‘Parris Island Cos’ (romaine, sensitive, PIC), PI 171676a (leaf, tolerant, P17), and ‘Shining Star’ (leaf, sensitive, SHI). Although ‘Eruption’ and ‘Parris Island Cos’ belong to the Latin and romaine types, respectively, they were included as a comparison pair because these two horticultural types are similar.

### Plant growth conditions

Seeds were sown in sterilized sand in ~ 12 cm plastic pots lined with a single layer coffee filter. Distilled water was used for germination. Five seeds were sown per pot in greenhouse and seedlings were thinned to one per pot eight days after sowing. The pots were then transferred to a Conviron CMP6050 growth chamber (Conviron, Winnipeg, MB, Canada). Plants were grown in the growth chamber with the following growing conditions: a constant temperature of 20^0^ C; 12 h light/dark cycle; light intensity of 245 µmol m^2^s^− 1^; and a relative humidity ranging from 26% to 75%. Pots were arranged in insert trays holding 18 plants per tray, which were placed on “1020” shallow trays (49.82 cm x 25.85 cm x 2.35 cm) without drainage holes to allow watering plants from the bottom. Immediately after transferring to growth chamber, the control and salt-treatment plants were watered according to the procedures described below.

### Experimental treatments

Plants were grown under two treatment conditions: 1) control - Hoagland Modified Basal Salt (HMBS) mixture (H353; Phyto Technology Laboratories, Shawnee Mission, Kansas, USA) solution used for nutrition of the plants and 2) salt – HMBS mixture plus NaCl and CaCl_2_ used for plant nutrition and salt stress application. Salt-treated entries received the following concentrations of salt [salinity level indicated by electrical conductivity (EC)] along with the HMBS mixture:

- 10 mM NaCl + 5 mM CaCl_2_ + HMBS (EC = 4570 µS cm^-1^) – first 9 days
- 20 mM NaCl + 10 mM CaCl_2_ + HMBS (EC = 6850 µS cm^-1^) – next 8 days
- 30 mM NaCl + 15 mM CaCl_2_ + HMBS (EC = 8870 µS cm^-1^) – last 24 days

Control plants received only HMBS mixture solution (EC = 2360 µS cm^− 1^).

Both the control and salt treatment solutions were prepared using distilled water. Salt stress was gradually introduced to avoid salt-shock to the plants. Trays were watered by reverse irrigation to avoid leaf injury from contact with salt solutions. During watering, 2 L of the respective solutions were applied to each tray and allowed to soak initially for 30 min and for 45 min when the plants grew bigger. Excess liquid was discarded from the trays. Plants were watered as needed with the objective to keep the sand moist so that the plants do not get salt shock.

In addition to salinity (EC) measurements of the HMBS mixture and salt solutions of different concentrations, the salinity of distilled water and sand used for the study were also measured using an EC/TDS Tester, HI98130 (HANNA Instruments, Smithfield, RI, USA). Salinity of distilled water and sand was 0.00 µS cm^− 1^ and 90 µS cm^− 1^, respectively.

### Experimental design

The study was conducted in a split-plot design with treatment as the main-plot factor and genotypes as the sub-plot factor with three replications. Each tray served as a unit for a main-plot factor (control or salt) facilitating application of the treatments, either control or salt to a whole tray containing the sub-plots (genotypes). Each experimental unit (sub-plot) consisted of two pots with a single plant in each pot.

### Measurements

**Fig. 1:**
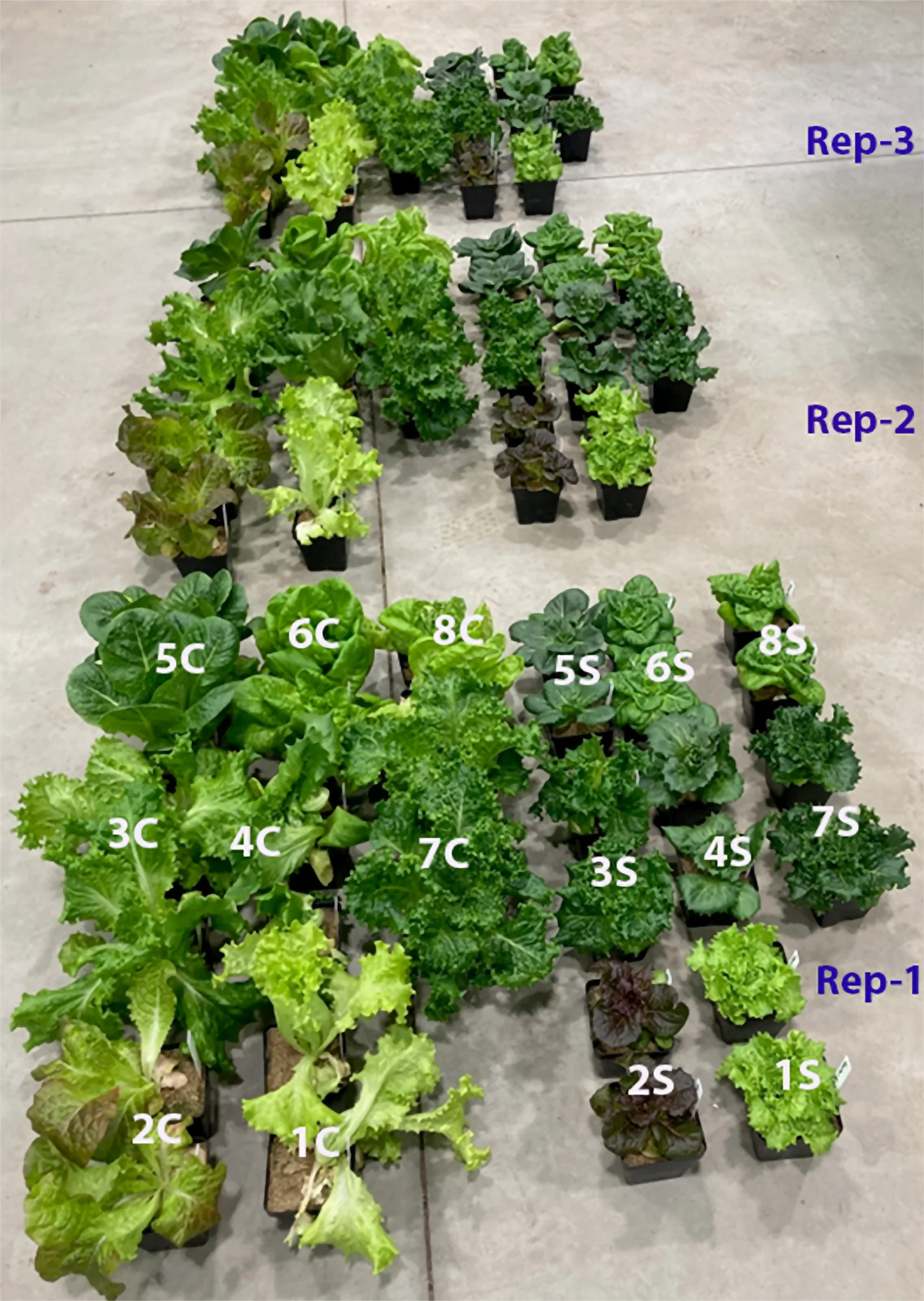
Experimental plants just before harvest for fresh weight and dry weight data collection. Two plants of each genotype per replication per treatment. (1C) LAU, (2C) ERU, (3C) SHI, (4C) EAB, (5C) PIC, (6C) MOR, (7C) p17, and (8C) MAY grown under control condition. 1S to 8S are corresponding genotypes grown under salt treatment

After 41 days of treatment application in the growth chamber, leaf chlorophyll index (SPAD) was measured on three intermediate-aged leaves per plant and averaged. A SPAD-502 m (Konica Minolta Sensing Inc., Tokyo, Japan), hand-held meter was used for this measurement. When optical methods for measuring leaf chlorophyll content are applied, index values (e.g. SPAD value) are commonly used to specify the relative leaf chlorophyll content, but not absolute chlorophyll content or concentration [63]. After measuring chlorophyll index the plants were harvested to measure shoot FW and dry weight (DW) (Fig. 1). Shoot FW were recorded immediately after harvest and shoot DW were recorded after drying the shoots for three days in drying oven at 70^0^ C.

### Lipid extraction

Approximately 100 mg fresh leaf tissue was collected from the third leaf from the apical meristem. Leaf tissue was collected 31 days after treatment applications. Tissue was placed in 1.5 ml microfuge tube, flash frozen in liquid nitrogen, placed on dry ice and shipped overnight to Kansas Lipidomic Research Center (KLRC, Kansas State University, Manhattan, KS, USA) for lipid and fatty acid extraction and quantification. At KLRC the protocol of Shiva et al. [64] was followed for lipid extraction. Briefly, frozen lettuce tissue was dropped into 1.0 ml preheated isopropanol, heated at 75° C for 15 min, and cooled to room temperature. Three ml chloroform: methanol: water (30/41.5/3.5, v/v/v) were added to each sample, and the samples were shaken at 100 rpm on an orbital shaker overnight. Each extract was transferred to a clean tube. The remaining solvent was removed from the extracted lettuce tissue by drying under a fume hood for a short time. The extracted tissue was dried in an oven at 105 °C overnight and weighed to express the lipid content on a dry weight basis.

### Lipid profiling

Lipid profiling was performed by the KLRC using an automated electrospray ionization-tandem mass spectrometry (ESI–MS/MS) approach. The lipid profiling was targeted since the lab did not have internal standards for all lipids. Analysis of the polar lipids were performed using the triple quadrupole mass spectrometer with a linear ion trap and an electrospray source, the Sciex 4000 QTRAP (SCIEX, Framingham, MA, USA) as described earlier [65]. The instrument parameters and the internal standards used for the polar lipids analysis are given in Table S1. Analysis of the TAGs were performed using the XEVO-TQS triple quadrupole mass spectrometer (Waters Corporation, Milford, MA, USA) as previously described [66]. The instrument parameters and the internal standards used for the TAGs analysis are given in Table S2. The lipid values were calculated as normalized mass spectral signal/milligram (mg) of leaf dry weight, where a value of 1 is the intensity of 1 nmol of internal standard (with an adjustment for variation in response with mass-charge ratio, m/z). For the diacyl or monoacyl phospholipids, the response of each compound was very close to the response of an internal standard of the same class. Thus, the numbers reported as normalized signal/mg leaf dry weight for diacyl or monoacyl phospholipids can be considered equal to nmol/mg leaf dry weight. However, for the galactolipids and TAGs studied, there is some variation in molar response among compounds within a class and/or in comparison to the internal standard. Additionally, in some cases, due to lack of an internal standard that closely matches the structure of the lipids in sample, a poorly matched standard may have been used. Thus, the normalized signal/mg leaf dry weight allows for a reasonably good comparison of the same lipid among samples but may provide only a rough indicator of the relative amount of that lipid compared to other compounds in the sample [67, 68]. Lipid data were also reported as mol% of the total lipids analyzed.

### Data quality control

Average and coefficient of variation (CV) from identical samples made from pooling samples and running several times were computed. If these averages were less than 0.002 nmol for polar lipids or 0.0005 nmol for TAGs, this lipid was considered below the limit of detection and removed from further analysis. Lipids with CV ≥ 0.3 were also removed from further analysis.

### Fatty acid analysis

Fatty acid analysis was performed at the KLRC using the same lipid samples described above following the methods described below:

#### Preparation of fatty acid methyl ester

Fatty acid methyl ester was prepared following the methodology described by Christie [69]. Briefly, for each sample, 50 nmol pentadecanoic acid (15:0; internal standard) and lipid extract (about 2 mg dry tissue weight) were combined in a screw-cap tube. The solvent was evaporated under a nitrogen stream. One ml 3 M methanolic hydrochloric acid was added and each sample was bubbled with nitrogen gas and the tube was capped. The samples were heated at 78 °C for 30 min and cooled to room temperature. Two ml water (Optima grade) were added, and each sample was extracted twice with 2.5 ml hexane: chloroform (4:1, v/v) each time, with vortexing and centrifuging to separate the phases. The combined organic phases (upper phases) were dried down completely under nitrogen gas, redissolved in 150 µl hexane, and transferred to a GC vial with insert, and one microliter was injected in a 6890 N Gas Chromatograph coupled to a flame ionization detector (GC-FID, Agilent Technologies, Santa Clara, CA, USA).

#### GC method

The analysis was carried out using the Agilent 6890 N GC-FID. The GC was fitted with a DB-23 capillary column (column length − 30 m, internal diameter − 250 μm, film thickness − 0.25 μm). Helium was used as the carrier gas at a flow rate of 1.5 mL/min. The GC oven temperature ramp was operated at an initial temperature of 100 °C, held 1 min, increased at 25 °C/min to 175 °C, then increased at 6 °C/min to 230 °C and held 5 min. The FID detector was operated at 260 °C, the hydrogen flow to the detector was 30 mL/min, air flow was 400 mL/min and make up gas helium flow was 25 ml/min. The data were processed using Agilent ChemStation software.

### Lipid unsaturation index

The unsaturation index of each lipid was calculated using the formula given below, which was used earlier by Hong et al. [70]

$$\sum\nolimits_{i=1}^n\left(\left(N_{i}* \mathrm{mol}\;\%_i\right)/100\right)$$

where n is the total number of lipid molecular species measured, *N* is the number of double bonds in the *i*th lipid molecular species, and mol % is the percentage (on a mol basis) of the *i*th lipid molecular species compared with the total lipids in the sample.

### Data analysis

For each trait, the sub-plot mean of the two plants within a replication was used for statistical analysis. Missing value of a single plant was imputed by the average of the other five plants of the same genotype and treatment. The percent change (increase/decrease) in each trait due to salt treatment was calculated as: percent change = ((value in salt-treated – value in control)/value in control) * 100). Analysis of variance (ANOVA) for the traits FW, DW, chlorophyll index, percent fatty acid, lipid content, lipid unsaturation, lipid ratio, and lipid molecular species were performed using the R software version 4.3.2 (2023-10-31 ucrt) [71]. Pearson’s correlation co-efficient and regression analyses were also performed using R, while Student’s t-test were performed using Excel software (Microsoft Co., Redmond, WA, USA). Unless otherwise noted, statistical differences were considered significant at *P* ≤ 0.05.

## Results

### Effect of salt treatment on lettuce fresh weight, dry weight and chlorophyll index

ANOVA indicated significant effects of treatment, genotype, and genotype-by-treatment (G x T) interaction on FW, DW and chlorophyll index (Table 1). The raw data for the three replications for these traits that were used for ANOVA is provided in Table S3. ANOVA for FW and DW were performed using natural log (Ln) transformed data since the original data did not give a normal distribution of the residuals. A significant G x T interaction indicated that different genotypes responded differently to the salt treatment for the trait.

**Table 1:** Analysis of variance of lettuce fresh weight, dry weight and chlorophyll index

Source^y^	Df	Mean Squares^z^		
		Fresh weight (Ln)	Dry weight (Ln)	Chlorophyll index (SPAD value)^x^
Replication	2	0.045	0.063	7.19
Treatment (T)	1	15.575***	3.514**	2108.80***
Error (a)	2	0.020	0.006	0.47
Genotype (G)	7	0.438***	0.727***	619.97***
G x T	7	0.050**	0.052**	11.09**
Error (b)	28	0.013	0.014	3.04

^z^Asterisks denote significance at *P* ≤ 0.01 (**), and *P* ≤ 0.001 (***)

^y^The study involving two treatments (control and salt stress) and eight genotypes was conducted in growth chamber

^x^Chlorophyll index data collected a day before fresh weight data were recorded

Compared to the control, the salt treatment significantly (*P* ≤ 0.05) reduced FW and DW and increased chlorophyll index for all genotypes (Table 2). Reduction in FW ranged from 58% for the salt-tolerant genotype ‘Laura’ to 74% for the salt-sensitive genotype ‘Parris Island Cos’. Similarly, the reduction in DW ranged from 24% for ‘Laura’ to 53% for ‘Parris Island Cos’. Chlorophyll index increased from 25% for ‘Parris Island Cos’ to 94% for ‘Laura’ (Table 2). Although overall FW loss was high and the differences between tolerant and sensitive genotypes were generally small, except within the crisphead pair ‘Laura’ at 58% loss vs. ‘Early Bird’ at 73% loss, each of the four genotype pairs maintained their relative ranking for salt tolerance.

**Table 2:** Effect of salt stress on lettuce fresh weight, dry weight and chlorophyll index

Genotypes	Fresh weight (FW, g)			Dry weight (DW, g)			Chlorophyll index (SPAD value)^x^		
	Control^y^	Salt	(%)^w^	Control	Salt	(%)	Control	Salt	(%)
LAU	35.1 ± 2.0 a^z^	14.7 ± 0.94 b	-58	1.7 ± 0.07 a	1.3 ± 0.10 b	-24	13.3 ± 0.50 a	25.8 ± 0.89 b	94
EAB	56.6 ± 4.1 a	15.2 ± 1.29 b	-73	2.4 ±0.23 a	1.5 ± 0.12 b	-36	32.7 ± 0.92 a	49.8 ± 1.84 b	52
MOR	45.9 ± 2.4 a	17.5 ± 0.80 b	-62	2.2 ± 0.07 a	1.5 ± 0.06 b	-30	25.8 ± 1.08 a	39.2 ± 0.71 b	52
MAY	39.7 ± 3.2 a	14.9 ± 0.67 b	-63	2.2 ± 0.24 a	1.4 ± 0.07 b	-35	15.7 ± 0.46 a	26.7 ± 0.43 b	70
P17	49.7 ± 2.3 a	15.9 ± 0.69 b	-68	3.3 ± 0.17 a	1.7 ± 0.10 b	-49	33.2 ± 2.22 a	49.1 ± 0.32 b	48
SHI	59.9 ± 2.9 a	16.8 ± 1.80 b	-72	3.7 ± 0.10 a	1.8 ± 0.20 b	-50	23.3 ± 0.89 a	38.9 ± 1.02 b	67
ERU	26.7 ± 3.2 a	7.9 ± 0.97 b	-71	1.4 ± 0.17 a	0.7 ± 0.08 b	-50	23.8 ± 0.87 a	33.6 ± 0.89 b	41
PIC	66.1 ± 2.1 a	17.2 ± 0.38 b	-74	4.4 ± 0.09 a	2.1 ± 0.01 b	-53	43.3 ± 0.79 a	54.0 ± 0.61 b	25

Means followed by different letters within each genotype (i.e., Control vs Salt) for each parameter indicate significant differenceat *P* ≤ 0.05 based on Student’s t-test

^x^Chlorophyll index data were recorded a day before FW data collection

^y^The study involving two treatments (control and salt stress) and eight genotypes was conducted in growth chamber

^z^Means followed by standard error (*n* = 3, two plants per replication; 6 biological replicates)

^w^% change (decrease/increase) due to salt treatment as compared to control

### Changes in fatty acids level under salt stress

A total of nine fatty acids, namely myristic, palmitic, stearic, oleic, linoleic, α-linolenic, arachidic, behenic, and lignoceric acids were detected and analyzed in the present study (Table S4). ANOVA on percent fatty acids indicated significant treatment effects for all the fatty acids except lignoceric acid (Table S5). The genotype effect was significant for all nine fatty acids. The G x T interaction effect was significant only for five fatty acids: myristic, palmitic, stearic, oleic, and arachidic (Table S5).

**Fig. 2:**
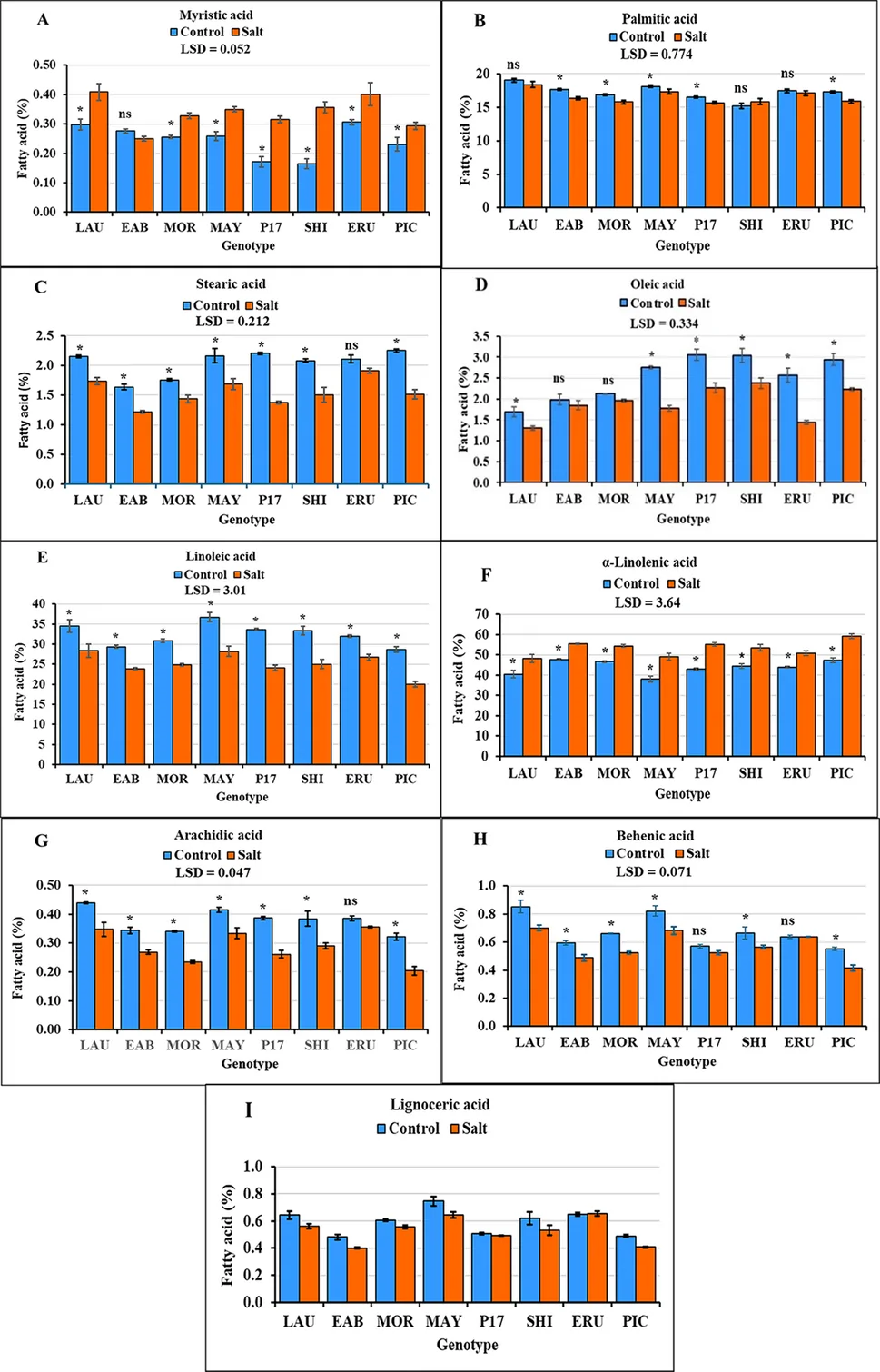
Bar graphs showing changes under salt stress in nine fatty acids for eight lettuce genotypes. **A** Myristic acid, (**B**) Palmitic acid, (**C**) Stearic acid, (**D**) Oleic acid, (**E**) Linoleic acid, (**F**) α-Linolenic acid, (**G**) Arachidic acid, (**H**) Behenic acid, and (**I**) Lignoceric acid. Error bars represent standard error; * represents significant difference between control and salt treatment means based on LSD (≤ 0.05); ns represents non-significant difference

In general, myristic acid and α-linolenic acid levels increased, while the levels of the other seven fatty acids decreased in response to salt stress (Fig. 2; Table S6). Under control condition, the mean over eight genotypes was highest for α-linolenic acid (43.9%) followed by linoleic acid (32.4%) and palmitic acid (17.3%). On the other hand, the lowest overall mean was for myristic acid (0.25%) followed by arachidic acid (0.38%) and lignoceric acid (0.59%). Under salt stress, the ranking of the nine fatty acids based on overall mean remained the same as control except the lowest and second lowest ranks were interchanged, with overall mean value ranging from 53.2% for α-linolenic acid to 0.29% for arachidic acid (Table S6). Based on the least significant difference (LSD), the differences in mean myristic acid level between control and salt treatment were significant for all genotypes except ‘Early Bird’, with increase in myristic acid due to salt treatment ranging from 27% for ‘Parris Island Cos’ to 117% for ‘Shining Star’ (Fig. 2A; Table S6). Significant differences in mean α-linolenic acid level between control and salt treatment were observed for all eight genotypes, and the increase ranged from 16% for ‘Early Bird’ and ‘Eruption’ to 29% for ‘Mayfair’ (Fig. 2F; Table S6).

For palmitic acid, significant differences between treatment means were observed for five genotypes with control means being higher and the reduction in palmitic acid level due to salt treatment ranged from 4% in ‘Mayfair’ to 8% in ‘Parris Island Cos’ (Fig. 2B; Table S6). Mean stearic acid level between control and salt treatment differed significantly for all genotypes except ‘Eruption’, and the reduction in stearic acid level ranged from 18% in ‘Morgana’ to 38% in PI 171676a (Fig. 2C; Table S6). For oleic acid significant differences in treatment means were observed for six genotypes and the range in reduction in oleic acid level due to salt treatment ranged from 22% in ‘Shining Star’ to 44% in ‘Eruption’ (Fig. 2D; Table S6). Mean linoleic acid level between control and salt treatment differed significantly for all eight genotypes and the reduction in linoleic acid level ranged from 16% for ‘Eruption’ to 30% for ‘Parris Island Cos’ (Fig. 2E; Table S6). Significant differences in mean arachidic acid level between control and salt treatment were observed for all genotypes except ‘Eruption’, and the reduction in arachidic acid level ranged from 20% for ‘Mayfair’ to 37% for ‘Parris Island Cos’ (Fig. 2G; Table S6). Mean behenic acid level between control and salt treatment differed significantly for all genotypes except PI 171676a and ‘Eruption’ and the reduction in behenic acid level ranged from 15% for ‘Shining Star’ to 25% for ‘Parris Island Cos’ (Fig. 2H; Table S6). ANOVA indicated non-significant treatment effect on lignoceric acid level, however, based on trend in the data some reduction in this fatty acid level due to salt stress was observed in all genotypes except for ‘Eruption’ (Fig. 2I; Table S6).

### Correlation among changes in fatty acids, biomass and physiological traits under salt stress

Pearson’s correlation coefficients among the nine fatty acids, FW, DW, and chlorophyll index were computed using the data on percentage change due to salt treatment in each of the traits. Myristic acid had a significant positive correlation (*r* = 0.768*) with palmitic acid (Table S7). Stearic acid had significant positive correlations with linoleic acid (*r* = 0.870**) and arachidic acid (*r* = 0.784*), while linoleic acid also had a significant positive correlation (*r* = 0.744*) with arachidic acid and a significant negative correlation (*r* = -0.773*) with α-linolenic acid (Table S7). A significant positive correlation (*r* = 0.848**) was observed between behenic acid and lignoceric acid. No significant correlation of any of the fatty acids with FW, DW or chlorophyll index was observed.

### Composition of the lettuce leaf lipidome

**Fig. 3:**
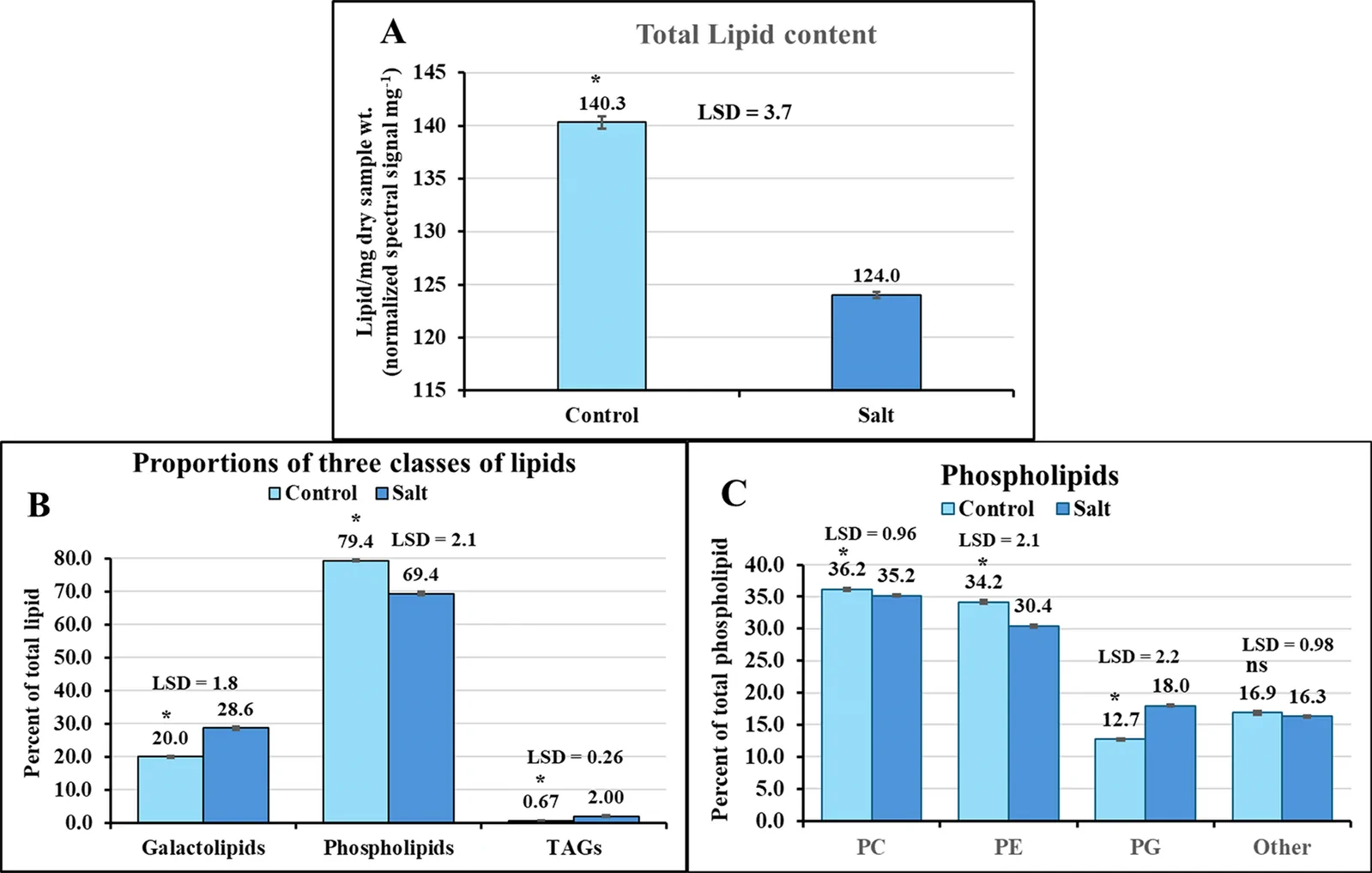
Bar graphs showing components of lettuce leaf lipidome under control and salt treatment. **A** total lipid content, (**B**) proportions of three classes of lipids (galactolipids, phospholipids, TAGs), and (**C**) proportions of phospholipids PC, PE, PG and the total of the other five phospholipids. The lipid data was collected from three replications of each genotype per treatment, and each replication data was average of two plants. Error bars represent standard error; * represents significant difference between control and salt treatment means based on LSD (≤ 0.05); ns represents non-significant difference

A total of 295 different lipid species were detected from the lettuce leaf samples, however, after data quality control (Methods), 130 lipid species remained and were used for further analyses in the present study (Tables S8 and S9). These lipid species belong to 15 different lipid classes, namely, galactolipids MGDG and DGDG; phospholipids PG, PC, PE, phosphatidylinositol (PI), phosphatidylserine (PS), PA; lysophosphoilipids lysophosphatidylcholine (LysoPC) and lysophosphatidylethanolamine (LysoPE); and five TAGs with different numbers of acyl chains, i.e., 16:0, 18:3, 18:2, 18:1, and 18:0. Under control condition the mean of total lipid content for the eight genotypes was 140.3 (normalized signal per mg dry sample weight), which significantly reduced to 124 due to salt stress (Fig. 3A). The composition of the mean lipid content over all eight lettuce genotypes was 79% phospholipids, 20% galactolipids, and ~ 1% TAGs. Under salt stress, the phospholipids significantly reduced to 69%, while the galactolipids and TAGs increased significantly to 29% and 2%, respectively (Fig. 3B). Among the eight phospholipids, under control condition the mean lipid content over the eight genotypes were 36.2% PC, 34.2% PE, 12.7% PG, and 16.9% others, i.e., the total of other five phospholipids in this study. Under salt stress PC and PE significantly reduced to 35.2% and 30.4%, respectively, while PG increased to 18% and the change in the total of other five phospholipids was non-significant (Fig. 3C).

### Membrane lipid remodeling under salt stress

ANOVA on lipid content/level based on normalized spectral signal per mg of sample dry weight indicated significant effect of treatment for all 15 lipid classes analyzed except PG, while genotype effect was significant for all 15 lipid classes (Table S10). The G x T interaction effect was significant only for DGDG and for all five TAGs analyzed. ANOVA on lipid unsaturation level indicated significant treatment and genotype effect for all 15 lipid classes, while G x T interaction effect was significant for DGDG, PG, LysoPE, PI, and for the five TAGs (Table S11). Two of the frequently studied lipid proportion changes due to salt stress are the ratios of DGDG:MGDG and PC:PE. In the present study, based on ANOVA treatment effect was significant for the PC:PE ratio but it was non-significant for the DGDG:MGDG ratio (Table S12).

#### Galactolipid content increased under salt stress

**Fig. 4:**
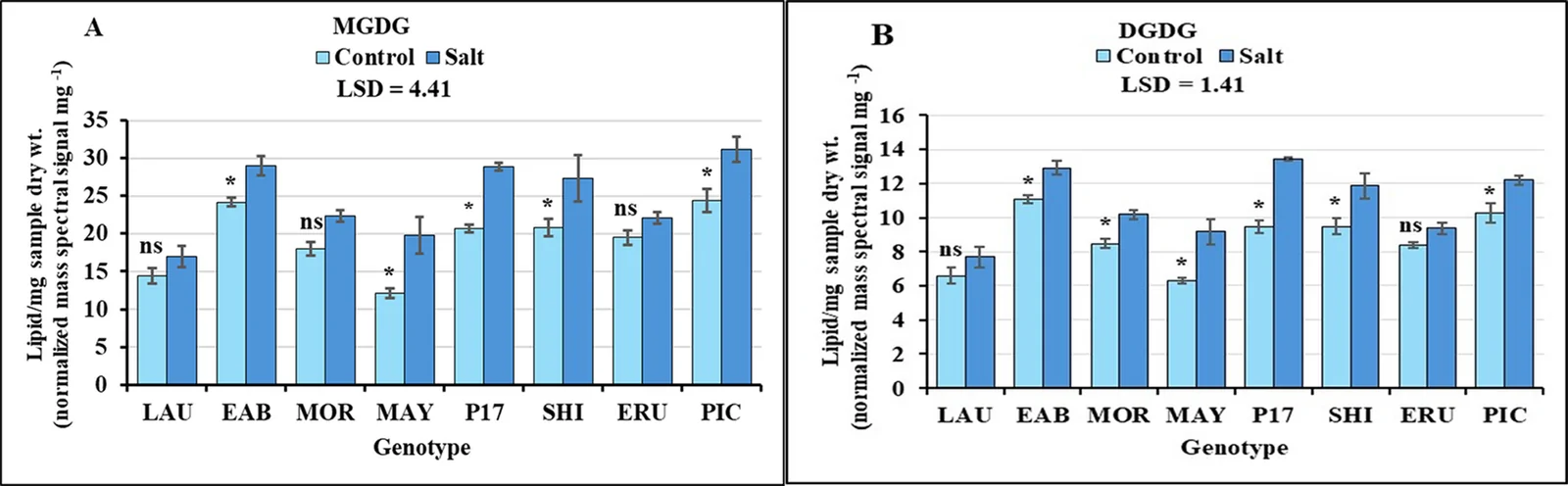
Changes in the galactolipids due to salt stress for the eight lettuce genotypes. **A** MGDG and (**B**) DGDG. Error bars represent standard error; * represents significant difference between control and salt treatment means based on LSD (≤ 0.05); ns represents non-significant difference

Both the galactolipids MGDG and DGDG increased in response to salt stress. Significant differences in mean lipid content between control and salt treatment were observed for all genotypes for both MGDG and DGDG except for ‘Laura’, ‘Morgana’, and ‘Eruption’ for MGDG and for ‘Laura’ and ‘Eruption’ for DGDG (Fig. 4A, B; Table S13). The increase in MGDG content ranged from 20% for the genotype ‘Early Bird’ to 64% for ‘Mayfair’, while the increase in DGDG content ranged from 16% for ‘Early Bird’ to 46% for ‘Mayfair’ (Table S13).

#### Content of most phospholipids decreased under salt stress

**Fig. 5:**
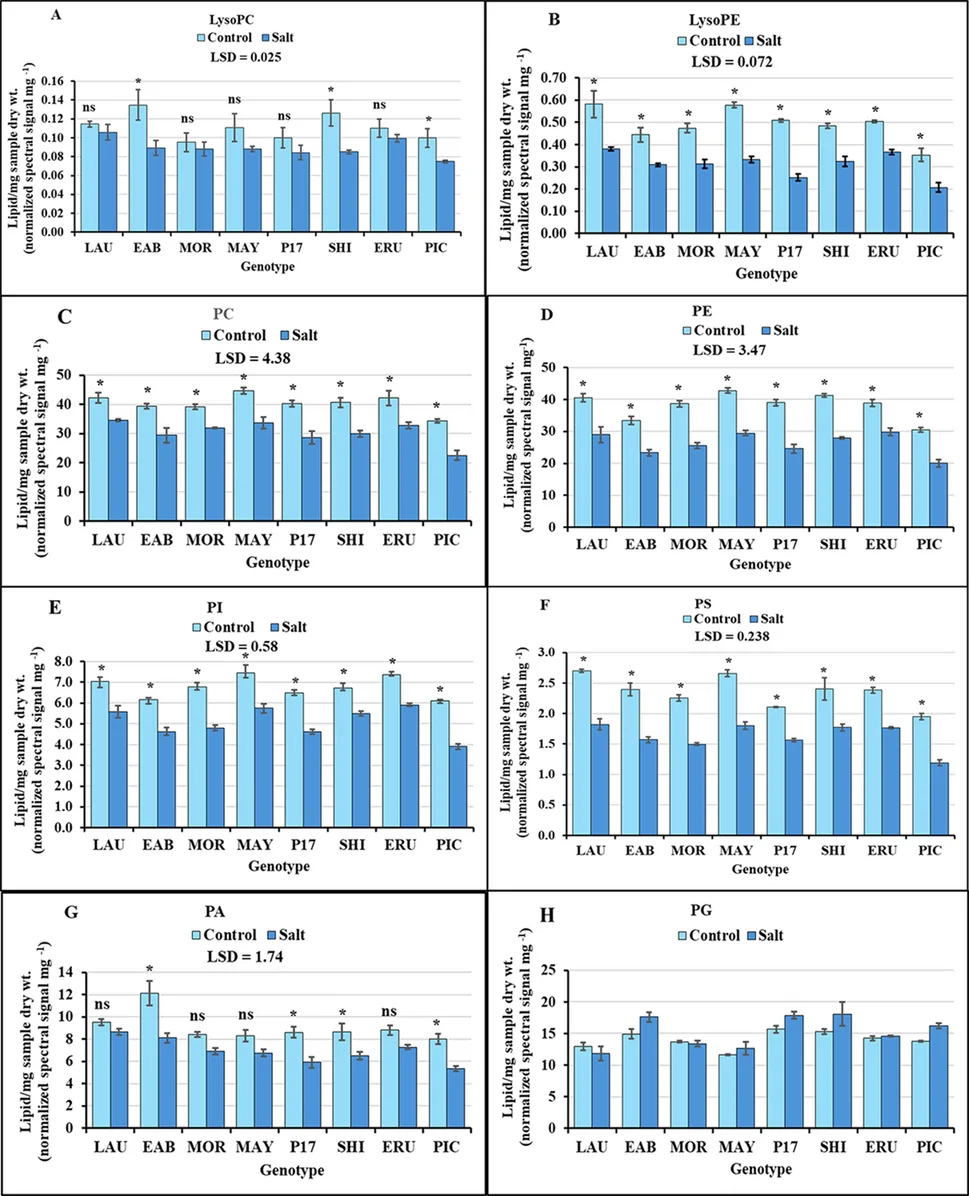
Changes in phospholipids due to salt stress for the eight lettuce genotypes. **A** LysoPC, (**B**) LysoPE, (**C**) PC, (**D**) PE, (**E**) PI, (**F**) PS, (**G**) PA, and (**H**) PG. Error bars represent standard error; * represents significant difference between control and salt treatment means based on LSD (≤ 0.05); ns represents non-significant difference

The content of seven of the phospholipids (i.e., LysoPC, LysoPE, PC, PE, PI, PS, and PA) analyzed in this study, decreased in response to salt stress (Fig. 5; Table S13). The mean LysoPC content differed significantly between control and salt treatment for three of the four salt-sensitive genotypes ‘Parris Island Cos’, ‘Shining Star’, and ‘Early Bird’ with lipid content decrease of 25%, 33%, and 34%, respectively (Fig. 5A; Table S13). However, for all the four salt-tolerant genotypes the decrease in LysoPC level was not significant and the levels of decrease were lower than that of all four salt-sensitive genotypes indicating that the changes in LysoPC was possibly related to salt tolerance. Treatment means for lipid content differed significantly for all eight genotypes for each of the phospholipids LysoPE, PC, PE, PI, and PS (Fig. 5B, C, D, E, F; Table S13). The decrease in lipid content ranged from 27% for ‘Eruption’ to 50% for the genotype PI 171676a for LysoPE, from 18% for ‘Laura’ to 34% for ‘Parris Island Cos’ for PC, from 23% for ‘Eruption’ to 37% for PI 171676a for PE, from 18% for ‘Shining Star’ to 36% for ‘Parris Island Cos’ for the phospholipid PI, and from 26% for the genotypes PI 171676a, ‘Shining Star’, and ‘Eruption’ to 39% for ‘Parris Island Cos’ for PS (Table S13). Significant differences in treatment means for PA content were observed for the four genotypes ‘Early Bird’, PI 171676a, ‘Shining Star’, and ‘Parris Island Cos’ with decrease in PA content of 33%, 32%, 25%, and 33%, respectively (Fig. 5G; Table S13). As indicated by ANOVA (Table S10), the salt treatment effect was not significant for the phospholipid PG. However, observing the trend in data, although the differences between control and salt treatment were non-significant, PG increased in six genotypes and reduced slightly in the genotypes ‘Laura’ and ‘Morgana’ (Fig. 5H; Table S13).

#### TAG content increased under salt stress

**Fig. 6:**
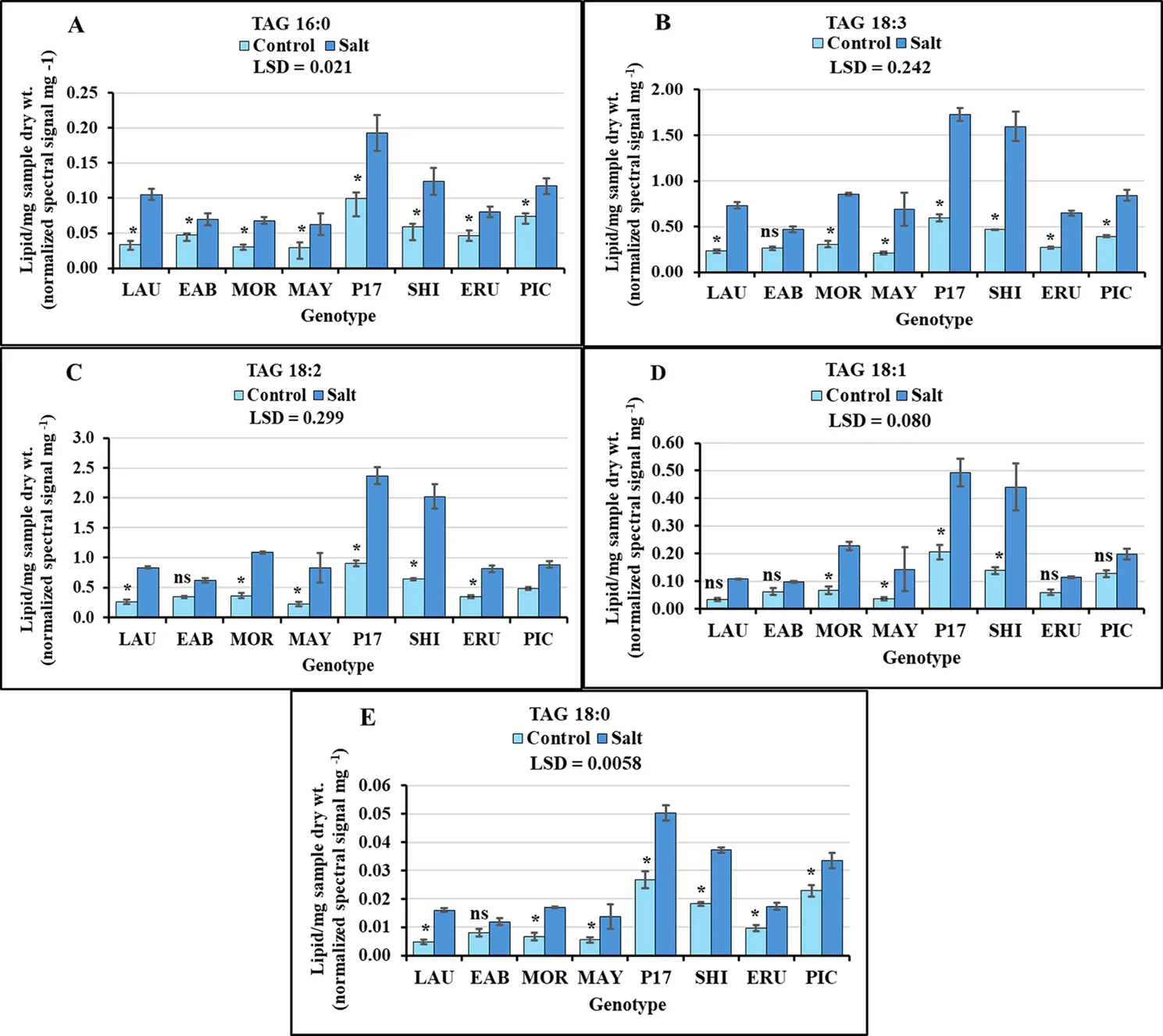
Changes in triacylglycerols of eight lettuce genotypes under salt stress. **A** TAG 16:0 (**B**) TAG 18:3, (**C**) TAG 18:2, (**D**) TAG 18:1, and (**E**) TAG 18:0. Error bars represent standard error; * represents significant difference between control and salt treatment means based on LSD (≤ 0.05); ns represents non-significant difference

Substantial increases in all five TAGs analyzed were observed in response to salt stress (Fig. 6; Table S13). The treatment means for the TAG 16:0 content differed significantly between control and salt treatment for all eight genotypes in the study. The increase ranged from 47% for ‘Early Bird’ to 214% for ‘Laura’ (Fig. 6A; Table S13). Significant differences between treatment means were observed for both TAG 18:3 and TAG 18:2 contents for all genotypes except ‘Early Bird’ (Fig. 6B, C; Table S13). The increase for TAG 18:3 content ranged from 113% for ‘Parris Island Cos’ to 242% for ‘Shining Star’, while the increase for TAG 18:2 content ranged from 82% for ‘Parris Island Cos’ to 275% for ‘Mayfair’. Significant differences between treatment means were observed for TAG 18:1 content for the genotypes ‘Morgana’, Mayfair’, PI 171676a, and ‘Shining Star’ with increase in the lipid level of 238%, 290%, 141%, and 219%, respectively (Fig. 6D; Table S13). Differences in treatment means between control and salt treatment for TAG 18:0 content were significant for all genotypes except ‘Early Bird’ and the increase in lipid content ranged from 47% for ‘Parris Island Cos’ to 229% for ‘Laura’ (Fig. 6E; Table S13).

#### Galactolipid unsaturation level increased under salt stress

The differences between treatment means based on LSD of unsaturation level for both MGDG and DGDG were significant for all eight genotypes. The increase in unsaturation level ranged from 31% for ‘Eruption’ to 86% for ‘Mayfair’ for MGDG, while for DGDG the increase ranged from 28% for ‘Eruption’ to 59% for ‘Mayfair’ (Table 3).

**Table 3:** Means ± SEs of lipid unsaturation level for the lettuce genotypes under control and salt stress

Genotype	MGDG			DGDG		
	Control	Salt	Change(%)^w^	Control	Salt	Change (%)
LAU	0.606 ± 0.047a^z^	0.833 ± 0.059 b	37	0.249 ± 0.020 a	0.331 ± 0.025 b	33
EAB	0.959 ± 0.013 a	1.294 ± 0.059 b	35	0.395 ± 0.001 a	0.514 ± 0.017 b	30
MOR	0.763 ± 0.045 a	1.097 ± 0.033 b	44	0.325 ± 0.013 a	0.446 ± 0.012 b	37
MAY	0.504 ± 0.032 a	0.935 ± 0.099 b	86	0.242 ± 0.007 a	0.384 ± 0.230 b	59
P17	0.804 ± 0.024 a	1.267 ± 0.052 b	58	0.335 ± 0.009 a	0.523 ± 0.014 b	56
SHI	0.795 ± 0.038 a	1.167 ± 0.091 b	47	0.327 ± 0.015 a	0.444 ± 0.008 b	36
ERU	0.781 ± 0.048 a	1.026 ± 0.038 b	31	0.305 ± 0.011 a	0.391 ± 0.016 b	28
PIC	1.041 ± 0.045 a	1.545 ± 0.091 b	48	0.383 ± 0.014 a	0.512 ± 0.010 b	34
LSD	0.174			0.048		

Genotype	PG			LysoPC		
	Control	Salt	Change(%)	Control	Salt	Change(%)
LAU	0.222 ± 0.013 a	0.258 ± 0.025 a	16	0.00145 ± 0.00003 a	0.00150 ± 0.00013 a	3
EAB	0.231 ± 0.011 a	0.337 ± 0.020 b	46	0.00175 ± 0.00015 a	0.00116 ± 0.00011 b	-34
MOR	0.245 ± 0.002 a	0.309 ± 0.011 b	26	0.00115 ± 0.00014 a	0.00123 ± 0.00015 a	7
MAY	0.188 ± 0.004 a	0.257 ± 0.016 b	37	0.00146 ± 0.00016 a	0.00127 ± 0.00008 a	-13
P17	0.236 ± 0.008 a	0.344 ± 0.022 b	46	0.00121 ± 0.00015 a	0.00109 ± 0.00010 a	-10
SHI	0.240 ± 0.009 a	0.350 ± 0.030 b	46	0.00152 ± 0.00021 a	0.00118 ± 0.00010 b	-22
ERU	0.234 ± 0.004 a	0.287 ± 0.010 a	23	0.00133 ± 0.00012 a	0.00134 ± 0.00005 a	1
PIC	0.242 ± 0.001 a	0.379 ± 0.013 b	57	0.00131 ± 0.00014 a	0.00108 ± 0.00003 a	-18
LSD	0.054			0.00033		

Genotype	LysoPE			PC		
	Control	Salt	Change(%)	Control	Salt	Change(%)
LAU	0.00636 ± 0.00042 a	0.00501 ± 0.00034 b	-21	1.075 ± 0.033 a	1.004 ± 0.027 a	-7
EAB	0.00438 ± 0.00029 a	0.00359 ± 0.00014 a	-18	0.930 ± 0.041 a	0.771 ± 0.044 b	-17
MOR	0.00556 ± 0.00024 a	0.00371 ± 0.00029 b	-33	1.010 ± 0.013 a	0.900 ± 0.013 a	-11
MAY	0.00637 ± 0.00019 a	0.00411 ± 0.00014 b	-35	1.166 ± 0.026 a	0.967 ± 0.073 b	-17
P17	0.00536 ± 0.00039 a	0.00276 ± 0.00004 b	-48	0.955 ± 0.013 a	0.735 ± 0.042 b	-23
SHI	0.00499 ± 0.00015 a	0.00382 ± 0.00056 b	-23	0.996 ± 0.048 a	0.789 ± 0.044 b	-21
ERU	0.00509 ± 0.00008 a	0.00445 ± 0.00006 a	-12	1.028 ± 0.049 a	0.883 ± 0.024 b	-14
PIC	0.00412 ± 0.00033 a	0.00255 ± 0.00021 b	-38	0.915 ± 0.006 a	0.688 ± 0.051 b	-25
LSD	0.00087			0.118		

Genotype	PE			PI		
	Control	Salt	Change(%)	Control	Salt	Change(%)
LAU	0.988 ± 0.222 a	0.818 ± 0.053 b	-17	0.121 ± 0.003 a	0.112 ± 0.0037 a	-7
EAB	0.768 ± 0.010 a	0.602 ± 0.019 b	-22	0.100 ± 0.001 a	0.086 ± 0.0028 b	-14
MOR	0.986 ± 0.031 a	0.715 ± 0.029 b	-27	0.120 ± 0.004 a	0.096 ± 0.0022 b	-20
MAY	1.071 ± 0.021 a	0.818 ± 0.032 b	-24	0.131 ± 0.005 a	0.112 ± 0.0034 b	-15
P17	0.902 ± 0.011 a	0.610 ± 0.016 b	-32	0.107 ± 0.003 a	0.083 ± 0.0009 b	-22
SHI	0.938 ± 0.017 a	0.690 ± 0.039 b	-26	0.109 ± 0.003 a	0.100 ± 0.0023 b	-8
ERU	0.911 ± 0.018 a	0.782 ± 0.027 b	-14	0.121 ± 0.001 a	0.110 ± 0.0004 b	-9
PIC	0.771 ± 0.012 a	0.548 ± 0.032 b	-29	0.111 ± 0.001 a	0.082 ± 0.0020 b	-26
LSD	0.093			0.0093		

Genotype	PS			PA		
	Control	Salt	Change(%)	Control	Salt	Change(%)
LAU	0.045 ± 0.001 a	0.036 ± 0.0009 b	-20	0.233 ± 0.005 a	0.245 ± 0.016 a	5
EAB	0.039 ± 0.001 a	0.029 ± 0.0005 b	-26	0.280 ± 0.020 a	0.213 ± 0.013 b	-24
MOR	0.040 ± 0.001 a	0.030 ± 0.0006 b	-25	0.211 ± 0.004 a	0.193 ± 0.009 a	-9
MAY	0.046 ± 0.001 a	0.035 ± 0.0011 b	-24	0.206 ± 0.013 a	0.186 ± 0.012 a	-10
P17	0.034 ± 0.001 a	0.028 ± 0.0003 b	-18	0.195 ± 0.012 a	0.146 ± 0.009 b	-25
SHI	0.038 ± 0.003 a	0.031 ± 0.0002 b	-18	0.200 ± 0.018 a	0.165 ± 0.015 a	-17
ERU	0.039 ± 0.001 a	0.033 ± 0.0002 b	-15	0.206 ± 0.011 a	0.190 ± 0.006 a	-8
PIC	0.035 ± 0.001 a	0.025 ± 0.0008 b	-29	0.206 ± 0.015 a	0.157 ± 0.008 b	-24
LSD	0.0038			0.040		

Genotype	TAG 16:0 acyl containing			TAG 18:3 acyl containing		
	Control	Salt	Change(%)	Control	Salt	Change(%)
LAU	0.00112 ± 0.00014 a	0.00418 ± 0.00044 b	273	0.01043 ± 0.00090 a	0.03885 ± 0.00301 b	272
EAB	0.00147 ± 0.00008 a	0.00257 ± 0.00030 a	75	0.01089 ± 0.00085 a	0.02315 ± 0.00197 b	113
MOR	0.00104 ± 0.00013 a	0.00262 ± 0.00014 b	152	0.01412 ± 0.00164 a	0.04433 ± 0.00132 b	214
MAY	0.00098 ± 0.00020 a	0.00235 ± 0.00034 b	140	0.00943 ± 0.00058 a	0.03483 ± 0.00833 b	269
P17	0.00307 ± 0.00017 a	0.00663 ± 0.00011 b	116	0.02422 ± 0.00146 a	0.08021 ± 0.00277 b	231
SHI	0.00184 ± 0.00012 a	0.00421 ± 0.00030 b	129	0.01937 ± 0.00042 a	0.07374 ± 0.00488 b	281
ERU	0.00148 ± 0.00022 a	0.00292 ± 0.00021 b	97	0.01143 ± 0.00032 a	0.03182 ± 0.00119 b	178
PIC	0.00258 ± 0.00020 a	0.00480 ± 0.00028 b	86	0.01805 ± 0.00082 a	0.04446 ± 0.00307 b	146
LSD	0.00123			0.00996		

Genotype	TAG 18:2 acyl containing			TAG 18:1 acyl containing		
	Control	Salt	Change(%)	Control	Salt	Change(%)
LAU	0.00846 ± 0.00098 a	0.03103 ± 0.00165 b	267	0.00079 ± 0.00009 a	0.00287 ± 0.00020 b	263
EAB	0.00992 ± 0.00063 a	0.02122 ± 0.00159 b	114	0.00129 ± 0.00021 a	0.00231 ± 0.00011 a	79
MOR	0.01169 ± 0.00130 a	0.03944 ± 0.00110 b	237	0.00149 ± 0.00027 a	0.00544 ± 0.00019 b	265
MAY	0.00706 ± 0.00108 a	0.02935 ± 0.00790 b	316	0.00084 ± 0.00011 a	0.00329 ± 0.00114 b	292
P17	0.02554 ± 0.00143 a	0.07636 ± 0.00353 b	199	0.00402 ± 0.00036 a	0.01076 ± 0.00017 b	168
SHI	0.01856 ± 0.00081 a	0.06504 ± 0.00433 b	250	0.00267 ± 0.00016 a	0.00907 ± 0.00096 b	240
ERU	0.01023 ± 0.00059 a	0.02801 ± 0.00162 b	174	0.00103 ± 0.00017 a	0.00276 ± 0.00007 b	112
PIC	0.01415 ± 0.00161 a	0.03346 ± 0.00216 b	136	0.00294 ± 0.00027 a	0.00521 ± 0.00027 b	77
LSD	0.00872			0.00139		

TAG 18:0 acyl containing						
Genotype	Control			Salt		Change(%)
LAU	0.00022 ± 0.00004 a			0.00083 ± 0.00003 b		277
EAB	0.00036 ± 0.00006 a			0.00061 ± 0.00007 b		69
MOR	0.00031 ± 0.00006 a			0.00090 ± 0.00005 b		190
MAY	0.00024 ± 0.00005 a			0.00070 ± 0.00020 b		192
P17	0.00117 ± 0.00012 a			0.00240 ± 0.00009 b		105
SHI	0.00080 ± 0.00001 a			0.00176 ± 0.00001 b		120
ERU	0.00044 ± 0.00006 a			0.00089 ± 0.00005 b		102
PIC	0.00113 ± 0.00011 a			0.00183 ± 0.00012 b		62
LSD	0.00025					

Means followed by different letters within each genotype (i.e., Control vs Salt) for each parameter indicate significant difference at *P* ≤ 0.05 based on LSD

^z^Means followed by standard error (n=3, two plants per replication; 6 biological replicates)

^w^% change (decrease/increase) due to salt treatment as compared to control

#### Unsaturation level of most phospholipids decreased under salt stress

In general, the level of unsaturation of all the phospholipids studied, except PG, decreased in response to salt stress (Table 3). For LysoPC, significant differences between treatment means for unsaturation level were observed only for the genotypes ‘Early Bird’ and ‘Shining Star’ with a 34% decrease for ‘Early Bird’ and a 22% decrease for ‘Shining Star’. Treatment means for unsaturation level differed significantly for all genotypes except ‘Early Bird’ and ‘Eruption’ for LysoPE, with decrease in unsaturation level ranging from 21% for ‘Laura’ to 48% for PI 171676a, while treatment means for lipid unsaturation level for PC differed significantly for all genotypes except ‘Laura’ and ‘Morgana’ and the decrease ranged from 14% for ‘Eruption’ to 25% for PI 171676a (Table 3). Significant differences between treatment means for lipid unsaturation level were observed for all eight genotypes for the lipids PE and PS with decrease ranging from 14% to 32% for PE and 15% to 29% for PS (Table 3). For the lipid PI, differences between treatment means for unsaturation level were significant for all genotypes except ‘Laura’ and the decrease ranged from 8% for ‘Shining Star’ to 26% for ‘Parris Island Cos’, while the differences between treatment means for the phospholipid PA were significant for the genotypes ‘Early Bird’, PI 171676a, and ‘Parris Island Cos’ with 24%, 25%, and 24% decrease, respectively (Table 3). The mean unsaturation level of PG differed significantly between control and salt treatment for all genotypes except ‘Laura’ and ‘Eruption’, and the increase in unsaturation level ranged from 26% for ‘Morgana’ to 57% for ‘Parris Island Cos’ (Table 3).

#### TAG unsaturation level increased under salt stress

As compared to the control, the unsaturation level of all five TAGs substantially increased under salt stress (Table 3). Treatment means for unsaturation level differed significantly for all eight genotypes for TAG 18:3, TAG 18:2, and TAG 18:0 with increase in lipid unsaturation level ranging from 113% for ‘Early Bird’ to 281% for ‘Shining Star’ for TAG 18:3, from 114% for ‘Early Bird’ to 316% for ‘Mayfair’ for TAG 18:2, and from 62% for ‘Parris Island Cos’ to 277% for ‘Laura’ for TAG 18:0 (Table 3). For both TAG 16:0 and TAG 18:1 significant difference between treatment means were observed for all genotypes except ‘Early Bird’, with an increase in unsaturation level ranging from 86% for ‘Parris Island Cos’ to 273% for ‘Laura’ for TAG 16:0 and from 77% for ‘Parris Island Cos’ to 292% for ‘Mayfair’ for TAG 18:1 (Table 3).

#### DGDG:MGDG ratio of lipid content

The MGDG and DGDG play major roles in determining the physicochemical properties of thylakoid membranes [72]. A stable MGDG/DGDG ratio in thylakoid membranes appears to be crucial for the stability and functional integrity of photosynthetic membranes [73]. In the present study, ANOVA indicated that the salt treatment did not have a significant effect on the DGDG/MGDG ratio (Table S12) thus the ratio was stable under salt stress.

#### PC:PE ratio of lipid content

**Fig. 7:**
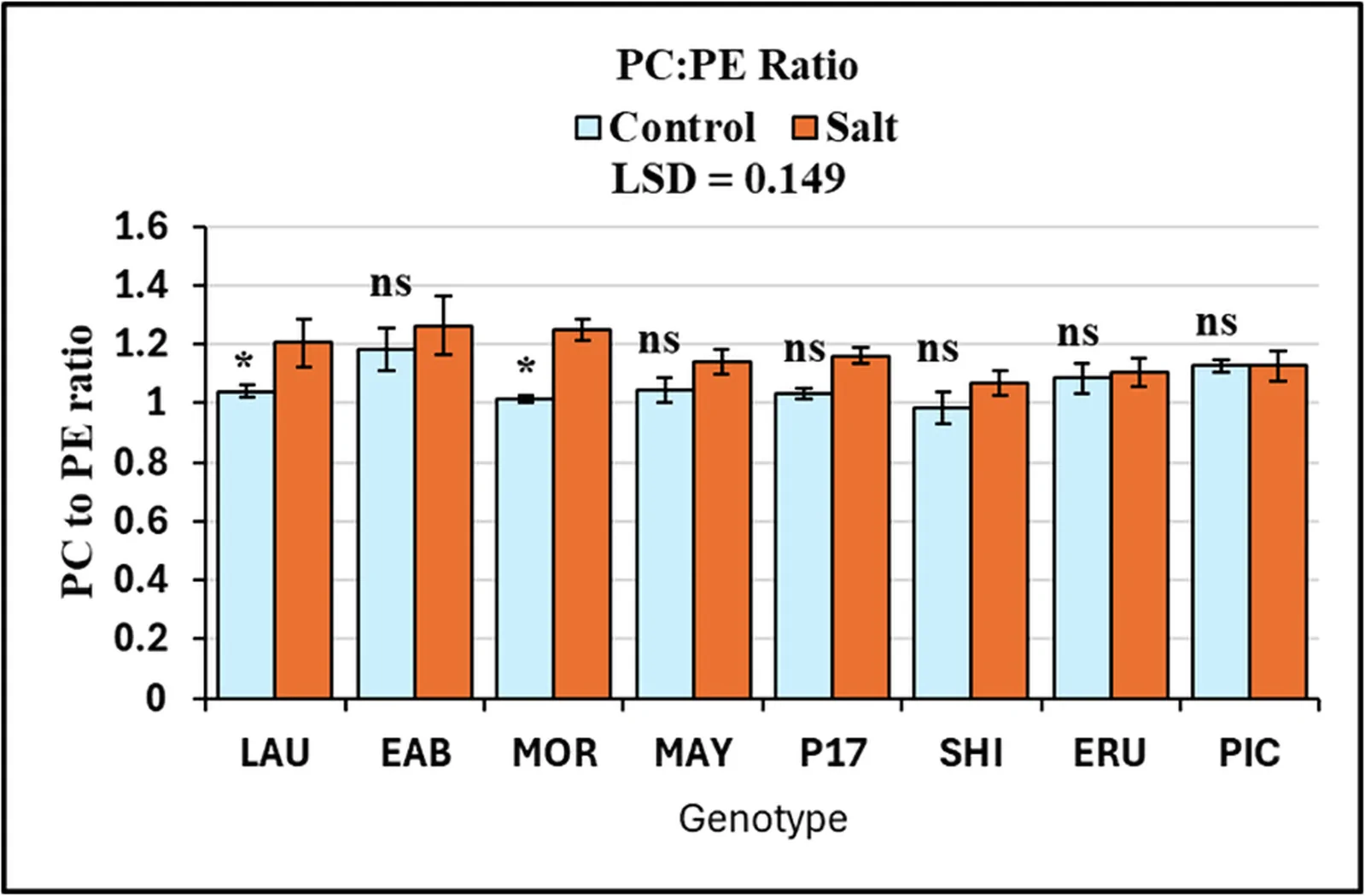
Bar graph showing changes in PC:PE ratio in lettuce due to salt stress. Error bars represent standard error; * represents significant difference between control and salt treatment means based on LSD (≤ 0.05); ns represents non-significant difference

The PC:PE ratio significantly increased in the two salt-tolerant genotypes ‘Laura’ (16% increase) and ‘Morgana’ (23% increase) over control (Fig. 7). For the rest of the genotypes, although non-significant, the PC:PE ratio increased from 2% to 12%, except for ‘Parris Island Cos’ where a less than 1% decrease was observed. A significant positive correlation (*r* = 0.776*) was observed between changes in PC:PE ratio and changes in FW due to salt stress indicating that PC:PE ratio of membrane lipids had a role in salt tolerance in lettuce in the present study.

### Lipid remodeling at the molecular species level

**Fig. 8:**
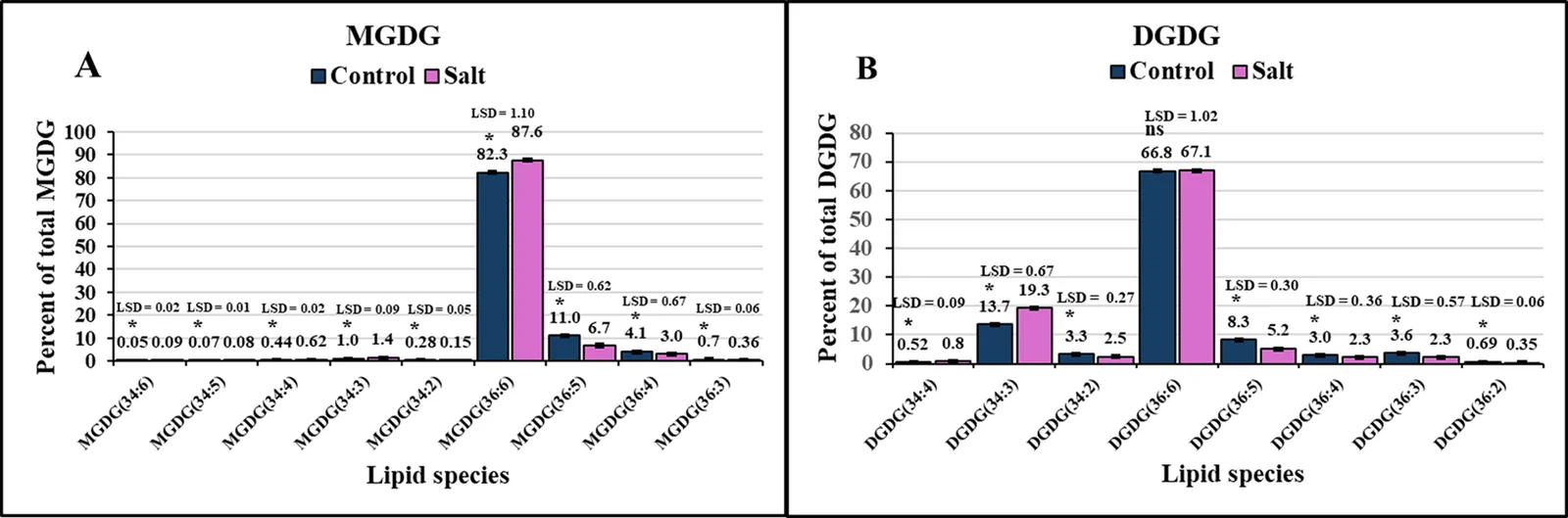
Changes in lipid molecular species in lettuce under salt stress. **A** Molecular species of MGDG and (**B**) molecular species of DGDG. Error bars represent standard error; * represents significant difference between control and salt treatment means based on LSD (≤ 0.05); ns represents non-significant difference

Lipid contents (normalized spectral signal per mg sample dry wt.) were analyzed for the molecular species of the 15 major lipid classes. ANOVA indicated significant treatment effect on 116 of the 130 lipid molecular species analyzed (Table S14). All nine molecular species for MGDG had significant changes (increase/decrease) due to salt stress, although seven of the molecular species had less than 5% of total lipid content for this lipid class (Fig. 8A). On the other hand, for DGDG seven of the eight molecular species had significant change due to salt stress, while for the molecular species C36:6 with the highest lipid percentage, the change was non-significant (Fig. 8B).

Proportion-wise, MGDG had 82% and 88% of C36:6 and 11% and 7% of C36:5 under control and salt stress conditions, respectively. For DGDG, C36:6 was ~ 67% under both control and salt stress conditions, while C36:5 was ~ 8% and ~ 5%; and C34:3 was ~ 14% and ~ 19%, respectively, under control and salt stress (Table S15). Thus, for both MGDG and DGDG, under control and salt stress conditions the predominant molecular species was C36, indicating that the galactolipid synthesis in lettuce follows the ER pathway [21]. Our results are in congruence with those of Mongrand et al. [74] that lettuce is an 18:3 plant.

For the phospholipids PC, PE, PG, PI, and PA, C34:2 was the major molecular species, and due to salt stress, it decreased by 18%, 31%, 18%, 27%, and 22%, respectively, for the five lipid classes. On the other hand, for the TAGs 16:0, 18:3, 18:2, 18:1, and 18:0, the major molecular species were C52:4, C54:7, C54:5, C54:3, and C56:8 and corresponding increases of 70%, 198%, 182%, 146%, and 90% were observed under salt stress (Table S15).

Percentage changes of lipid content of each of the molecular species for each of the genotypes is presented in Table S16. For some of the molecular species, differences in changes due to salt stress were observed between salt-tolerant and salt-sensitive genotypes. For example, for the major molecular species C36:6 in MGDG, the increase in lipid content was less in three of the salt-tolerant genotypes as compared to the corresponding salt-sensitive genotypes (Table S16). Similar trends in increase for the molecular species C32:1 and C34:4 of PG were also observed. On the other hand, the molecular species C34:2 for both PC and PA declined less in three of the salt-tolerant genotypes as compared to their corresponding sensitive genotypes (Table S16). Thus, these specific lipid molecular species had possible roles in salt tolerance in lettuce in the present study.

### Correlations among changes in lipid content, lipid unsaturation, biomass and physiological traits under salt stress

Pearson’s correlation coefficients among lipid contents (signal per mg dry sample weight) of the 15 lipid classes, FW, DW and chlorophyll index were computed using the data on percentage change due to salt treatment in each of the traits. The correlation coefficients indicated significant associations among several lipid classes as well as associations of the lipid classes with FW, DW, and chlorophyll index (Table 4). FW had significant positive correlations with DW, chlorophyll index, the lipids PC, PA, TAG 16:0, TAG 18:2, TAG 18:1, and TAG 18:0 and a significant negative correlation with PG, while DW had significant positive correlations with chlorophyll index, PC, and TAG 18:0 (Table 4). Chlorophyll index had significant positive correlations with TAG 16:0, TAG 18:2, TAG 18:1, and TAG 18:0 indicating possible role of TAG accumulation in lettuce leaves to maintain membrane stability and function of the photosynthetic apparatus. A significant positive correlation (*r* = 0.933**) between the galactolipids MGDG and DGDG was observed. This strong positive correlation may indicate possible coordinated metabolism of these two lipids.

**Table 4:** Correlation among percentage changes of lettuce lipid content, fresh weight, dry weight and chlorophyll index

	DW	Chl_ind	MGDG	DGDG	PG	LysoPC	LysoPE	PC	PE
FW	0.826**	0.731*	0.192	0.215	-0.840**	0.701	-0.123	0.725*	0.026
DW	-	0.711*	-0.017	-0.058	-0.701	0.389	0.191	0.773*	0.128
Chl_ind^x^		-	0.147	0.155	-0.529	0.187	0.100	0.645	0.191
MGDG			-	0.933**	0.267	-0.188	-0.674	-0.279	-0.428
DGDG				-	0.260	-0.097	-0.810*	-0.274	-0.549
PG					-	-0.878**	-0.275	-0.846**	-0.423
LysoPC						-	-0.012	0.597	0.230
LysoPE							-	0.532	0.767*
PC								-	0.495
		PI	PS	PA	TAG 16:0	TAG 18:3	TAG 18:2	TAG 18:1	TAG 18:0
FW		0.219	-0.033	0.801*	0.880**	0.584	0.723*	0.771*	0.950**
DW		0.216	-0.316	0.628	0.683	0.211	0.438	0.545	0.773*
Chl_ind		0.673	0.173	0.695	0.858**	0.684	0.722*	0.726*	0.850**
MGDG		-0.067	0.022	-0.086	0.015	0.500	0.583	0.537	0.103
DGDG		-0.076	0.234	-0.150	0.052	0.530	0.542	0.487	0.103
PG		-0.290	-0.019	-0.907**	-0.768*	-0.308	-0.441	-0.469	-0.825*
LysoPC		0.018	0.153	0.700	0.558	0.240	0.315	0.312	0.608
LysoPE		0.514	0.010	0.349	-0.027	-0.279	-0.176	-0.156	0.008
PC		0.580	0.187	0.842**	0.643	0.307	0.488	0.530	0.735*
PE		0.640	0.196	0.536	0.087	-0.139	-0.018	-0.113	0.124
PI		-	0.617	0.578	0.397	0.491	0.502	0.407	0.399
PS			-	0.163	0.073	0.449	0.305	0.170	0.035
PA				-	0.814*	0.558	0.677	0.663	0.874**
TAG 16:0					-	0.700	0.690	0.717*	0.973**
TAG 18:3						-	0.925**	0.884**	0.697
TAG 18:2							-	0.977**	0.768*
TAG 18:1								-	0.804*

Significance level, *P* ≤ 0.05 = 0.707, indicated by * in the Table and text; *P* ≤ 0.01 = 0.834, indicated by ** in the Table and text

^*x*^*Chl_ind* Chlorophyll index

The phospholipid PG had significant negative correlations with LysoPC, PC, PA, TAG 16:0, and TAG 18:0 (Table 4). PE had a significant positive correlation with LysoPE, while PC had significant positive correlations with PA and TAG 16:0. The phospholipid PA also had significant positive correlations with TAG 16:0 and TAG 18:0 (Table 4). TAG 16:0 had positive correlations with TAG 18:1 (*r* = 0.717*) and TAG 18:0 (*r* = 0.973**), while TAG 18:3 had positive correlations with TAG 18:2 (*r* = 0.925**) and TAG 18:1 (*r* = 0.884**). TAG 18:2 also had positive correlations with TAG 18:1 (*r* = 0.977**) and TAG 18:0 (*r* = 0.768*), whereas TAG 18:1 had a positive correlation with TAG 18:0 (*r* = 0.804*). The strong positive correlations among TAG 18:3, TAG 18:2, and TAG 18:1 may indicate coordinated metabolisms of these lipids or due to the regulation of their genes by the same transcription factor, WRINKLED1.

Pearson’s correlation coefficients were also computed among changes in lipid unsaturation levels of the 15 lipid classes, FW, DW and chlorophyll index using the data on percentage change due to salt treatment in each of the traits (Table S17). The correlation coefficients indicated similar associations among the traits as observed from the correlations among changes in lipid contents, FW, DW and chlorophyll index described above.

### Correlations between changes in lipid contents and fatty acid contents 

Most of the nine fatty acids studied belong as acyl chain groups to some of the 15 lipid classes in the present study, therefore, correlations between changes in fatty acids level/content and changes in lipid content/level due to salt treatment were also computed (Table S18). Both MGDG and DGDG contain alpha linolenic acid as acyl chains in their structures and significant positive correlations were observed between changes in alpha linolenic acid and changes in both MGDG and DGDG. The phospholipids LysoPE, PC, and PE had significant positive correlations with stearic acid and linoleic acid (Table S18). LysoPE also had a significant negative correlation with alpha linolenic acid, while PE also had a significant positive correlation with arachidic acid. The phospholipid PI had significant positive correlations with palmitic acid and arachidic acid, while PS had a positive correlation with behenic acid and TAG 18:3 had a significant positive correlation with myristic acid (Table S18).

### Regression analysis

Simple linear regression analysis between changes in lipid content and changes in FW due to salt stress with lipids as the predictor variables and FW as the response variable was also performed. The regressions were analyzed for the seven lipid classes (PG, PC, PA, TAG 16:0, TAG 18:2, TAG 18:1, and TAG 18:0) that had significant correlations with FW. The regression coefficients were significant for all seven regression models for the seven lipids, indicating significant relationships between changes in lipid content and changes in FW (Table 5). This confirms the significant relationships for these associations based on Pearson’s correlation (Table 4). The multiple *R*^2^ values were significant for all seven regressions (Table 5), and the percent variations in FW explained by the variations in lipids ranged from 52% for TAG 18:2 to 90% for TAG 18:0. Thus, the regression analysis indicated that variations in changes in FW due to salt treatment was influenced by changes in lipids and changes in FW can be predicted from changes in lipids, especially for the lipids PG, TAG 16:0, and TAG 18:0 with 71%, 78%, and 90% *R*^2^ values, respectively.

**Table 5:** Regression analysis of changes in lettuce lipid content and fresh weight due to salt stress

Fitted regression model	Overall regression significance		
	*R* ^2^	F (1, 6)	*P*-value
Based on lipid content			
FW = -63.56–0.480** (PG)	0.706	14.43	0.009
FW = -47.64 + 0.804* (PC)	0.525	6.64	0.042
FW = -55.35 + 0.530* (PA)	0.642	10.74	0.017
FW = -78.02 + 0.100** (TAG 16:0)	0.775	20.63	0.004
FW = -78.22 + 0.062* (TAG 18:2)	0.522	6.56	0.043
FW = -75.90 + 0.050* (TAG 18:1)	0.595	8.81	0.025
FW = -77.82 + 0.091*** (TAG 18:0)	0.903	55.58	0.0003

*, ** and *** indicate statistical significance at *P* ≤ 0.05, *P* ≤ 0.01 and *P* ≤ 0.001, respectively

### Genetic and breeding improvement potential for salt tolerance in lettuce based on knowledge of lipidomics

Correlations between lipid content of the genotypes under control condition and losses in FW due to salt stress were computed. Both MGDG and DGDG had significant negative correlation with changes in FW, suggesting that lower MGDG and DGDG containing genotypes had better salt tolerance (Table S19). On the other hand, LysoPE had a positive significant correlation with changes in FW, suggesting that higher LysoPE containing genotypes had better salt tolerance. Both MGDG and DGDG had significant negative correlations with most of the phospholipids, indicating that higher amount of MGDG and DGDG led to lower amount of those phospholipids (Table S19). Correlations between lipid content under control condition and lipid content under salt stress indicated strong positive correlation between MGDG under control and MGDG under salt (Table S20). Similarly strong positive correlation between DGDG under control and DGDG under salt was also observed (Table S20). This indicated that lettuce genotypes with lower MGDG and DGDG content under control condition had inherent ability to keep lower level of these two lipids under salt stress. Regression analysis was also performed between lipid content in the control genotypes and changes in FW due to salt treatment with the lipids as predictor variables and FW as the response variable (Table 6).

**Table 6:** Regression of MGDG and DGDG from control on fresh weight changes due to salt stress

Fitted regression model	Overall regression significance		
	*R* ^2^	F (1, 6)	*P*-value
Based on lipid content			
FW = -45.10–1.17** (MGDG_C)	0.735	16.62	0.007
FW = -41.83–2.94** (DGDG_C)	0.697	13.78	0.010

** indicate statistical significance at *P* ≤ 0.01

*MGDG_C* MGDG under control condition *DGDG_C* DGDG under control condition

The regression coefficients involving regressions of MGDG and DGDG content in control genotypes and changes in FW due to salt stress were significant indicating significant relationship between changes in FW and both MGDG and DGDG content in the control genotypes. The *R*^2^ values were also significant, indicating that both MGDG and DGDG give reliable estimates of losses in FW. The *R*^2^ values indicated that 74% and 70% variations in losses in FW were explained by MGDG and DGDG, respectively. Thus, using these regression models the FW losses could be predicted by MGDG and DGDG content in the control plants. The regression between LysoPE and changes in FW was not significant. Thus, optimizing the MGDG and DGDG content, fresh weight loss could be minimized or prevented in lettuce.

ANOVA involving the control genotypes indicated highly significant genotype mean squares for lipid content of all 15 lipid classes except LysoPC indicating that substantial genetic variations among the 8 lettuce genotypes exist for each of the 14 lipid classes (Table S21). Based on an average of 6 biological replicates per genotype the MGDG and DGDG content under control condition ranged from 12.1 to 24.2 and 6.3 to 11.1, respectively (Table S22). For the major phospholipids PC and PE, the ranges of lipid content were 39.3 to 42.2 and 33.4 to 42.8, respectively. Wide range of variation among the eight genotypes also existed for the five TAGs studied (Table S22). Thus, opportunities exist to breed salt tolerant lettuce cultivars by modulating desired lipid levels through genetic manipulation.

## Discussion

In this study, changes in fatty acids and lipids due to salt stress were analyzed and their correlations with changes in FW and DW were studied to determine the role of fatty acids and lipids in salt tolerance in lettuce. Associations between changes in chlorophyll index and changes in fatty acids and lipids due to salt stress were also studied since chlorophyll content is critical for efficient photosynthesis and serves as an indicator of overall plant health and productivity [75].

All eight genotypes had losses in FW and DW due to salt stress, however, the losses were less for the salt-tolerant genotypes as compared to the corresponding salt-sensitive genotypes (Table 2). Chlorophyll index increased in all eight genotypes, however, the increase was more in two salt-tolerant genotypes and less in the other two salt-tolerant genotypes as compared to their corresponding salt-sensitive genotypes (Table 2). Increase in chlorophyll index in response to salt stress in lettuce have also been reported in earlier studies [4, 22].

In general, the levels of myristic acid and α-linolenic acid increased in response to salt stress ranging from 27% to 117% and 16% to 29%, respectively, among the genotypes. On the other hand, the levels of the other seven fatty acids decreased in response to salt stress (Fig. 2; Table S6). While differences in changes in fatty acids between the salt-tolerant and salt-sensitive genotypes were observed, no significant correlation between changes in any of the fatty acids due to salt stress with changes in FW or DW was observed. Thus, although most of the fatty acids studied are acyl groups in the lipids studied, the fatty acids per se did not appear to have a role in salt tolerance in lettuce in the present study. Only a small effect of salt stress on fatty acids content was reported in roots of the relatively salt-tolerant and salt-sensitive barley genotypes, Sahara 3771 and Clipper, respectively [16]. The global lettuce leaf lipidome analysis based on the high-quality data set consisting of 130 different lipid molecular species from 15 major lipid classes in the present study showed that the average of the total lipid content across the eight genotypes decreased (11.4%) due to salt stress. A reduction in total lipid content in response to salt stress has been reported in leaves of tomato and in roots of barley. This reduction has been attributed to increased electrolyte leakage of membranes, indicating loss of membrane integrity [76, 77].

Plant photosynthesis is highly sensitive to salt stress, and the photosystems of plants can be severely damaged with increased duration and intensity of stress, leading to plant dehydration and death [78, 79]. MGDG and DGDG are the major lipids in the photosynthetic membranes and are involved in both photosynthesis and chloroplast development [80, 81]. Earlier studies in *Arabidopsis* have indicated that MGDG and DGDG play critical roles in photosynthetic efficiency and membrane stability [80, 82]. In the present study, based on the average lipid content across the eight genotypes, the leaf lipidome under control condition contained 20% galactolipids and increased to 29% under salt stress. Both MGDG and DGDG increased for all eight genotypes. However, the increases were non-significant for three and two of the salt tolerant genotypes, respectively, for MGDG and DGDG (Fig. 4A, B), indicating that the changes in the galactolipids were related to salt tolerance.

PG has a critical role in the function of photosystem II (PSII) in the thylakoid membranes. PG is known to be the only lipid completely essential for oxygenic photosynthesis, while the other plastidic lipids such as the MGDG and DGDG, found to be replaceable with glucolipids [83]. In our study, although the differences between control and salt treatment were not significant, PG increased in six genotypes and reduced slightly in the two salt-tolerant genotypes ‘Laura’ and ‘Morgana’, (Fig. 5H; Table S13). Increased PG content under salt stress was reported in leaves of salt cress and in the salt-tolerant buffalograss [54, 84].

Among all major phospholipids PC and PE are the predominant components (15% − 80%) in membranes [56]. The lettuce leaf lipidome in the present study consisted of 79% phospholipids, with PC (36%) and PE (34%) being the predominant ones among the eight phospholipid classes analyzed. Both PC and PE reduced significantly for all eight genotypes under salt stress. In response to salt stress, a decline in PC and PE in alfalfa [19] and total phospholipids and PC of the plasma membrane in winter wheat roots [57] were reported in earlier studies.

The increase in the plastidic galactolipids and decrease in most of the phospholipids outside of plastid in our study warrant a closer look into the biosynthesis of these glycerolipids, which has been reviewed in detail elsewhere [55, 85]. Fatty acids are exclusively synthesized within plastids, however, the assembly of fatty acids into the glycerolipids in the thylakoid membranes occurs through two distinct pathways, namely the prokaryotic pathway and the eukaryotic pathway. In the prokaryotic pathway, all steps in the glycerolipid biosynthesis take place within the chloroplast and thus this is also known as the plastidic pathway [55, 85]. Conversely, in the eukaryotic pathway, fatty acids are transported from the chloroplast to the cytosol for assembly into phospholipids in the endoplasmic reticulum (ER). Some of the phospholipids assembled in the ER can also return to the plastid to provide DAGs needed for the synthesis of galactolipids and SQDG in the plastid. MGDG synthesized through the eukaryotic pathway predominantly contains 18:3 fatty acids in the sn-2 position, thus these plants including lettuce are known as 18:3 plants [18]. The amount of phospholipid turnover to galactolipid is important, especially under stress conditions, since both classes of the lipids are needed to maintain membrane functionality under stress condition. In several 18:3 plant species, an increase in galactolipids and decrease in phospholipids have been reported under various abiotic stresses. In a study where lipid analyses conducted in maize leaves and roots under low-phosphate conditions showed an overall increase in non-phosphorus lipids (MGDG, DGDG and SQDG), and a decrease in phosphorus lipids (PC, PE, PI and PA), especially in leaf tissues [86]. Thus, under low-phosphorus stress, the degradation of phospholipids from eukaryotic pathway was enhanced to provide PA and DAG for the synthesis of MGDG, and then DGDG in the 18:3 plant maize. A phosphate-saving mechanism involving enhanced accumulation of DGDG under phosphate-deficient conditions has also been discovered, where the additional DGDG is exported to extra-plastidic membranes to partly replace phospholipids [72]. In another study, under saline-alkaline stress, created using 100 mmol NaHCO3 treatment, the level of PC decreased significantly, and a transcriptomic analysis showed an increased expression of genes encoding the enzymes phospholipase A and phospholipase D/non-specific phospholipase C, which suggested an activated PC turnover under saline–alkaline stress [87].

In rice, the OsMGD gene is highly expressed in photosynthetic tissues. It was found that OsMGD1 mediates membrane lipid remodeling to enhance photosynthesis and salt tolerance in rice seedlings under salt stress. Also, OsMGD1 increased chlorophyll content and photosynthetic efficiency, mitigating the impact of salt stress on biomass. Overexpression of OsMGD1 increased the content of galactolipids relative to wild type plants, while significantly reduced the content of phospholipids (PE and PI) [53]. These findings suggested that under salt stress, OsMGD1 might facilitate the synthesis of galactolipids by modulating the glycerolipid pathway between the cytosolic and plastidic compartments in the 18:3 plant rice.

Unlike oilseeds, plant leaves accumulate low levels of storage lipids such as TAG. While the galactolipids and phospholipids content may vary widely from plant species to species, the TAGs typically account for less than 1% of the total leaf lipids [88]. In the present study, based on the average across the eight genotypes, the leaf lipidome contained 0.67% TAGs and increased to 2% under salt stress. All five TAGs analyzed in this study increased substantially under salt stress (Fig. 6). One of the salt-tolerant genotypes (‘Laura’) consistently had about 4-fold higher percentage increase in all five TAGs over its corresponding salt-sensitive genotype, ‘Early Bird’ (Table S13). Increase in TAG and its contribution to salt tolerance was reported in alfalfa [19]. An increase in 18:3-containing TAGs under heat stress and its possible role in sequestering fatty acids during membrane lipid remodeling mechanism was also reported earlier [67].

The degree of unsaturation of the acyl chains inside the lipid bilayer is a major factor shaping membrane fluidity during stress [89, 90]. A higher unsaturation degree can lead to a more fluid state of the membrane since a cis-double bond creates a kink as steric hindrance in intermolecular package [91]. The C18 species of membrane fatty acids are prominent modulators of unsaturation degree [92]. For salt sensitive species, the increase and decrease in the unsaturation degree reflect defense and damage, respectively, depending on their sensitivity to certain salinity [90]. In some cases, higher degrees of unsaturation may also make the membrane more fluid and thereby more permeable, allowing Na^+^ and Cl^−^ ions to enter cells and thus having negative impact under salt stress, indicating higher membrane lipid unsaturation is not always beneficial for salt stress tolerance. For example, in maize (*Zea mays*), the salt-tolerant varieties exhibited a greater decline in lipid unsaturation than did the sensitive ones upon NaCl treatment [90]. In the present study, of the eight phospholipids analyzed, the unsaturation index increased for PG and decreased for rest of the phospholipids due to salt stress, however, the decrease was in general less for the salt-tolerant genotypes as compared to their corresponding sensitive genotypes (Table 3). Due to reduction in unsaturation, the 18:3 acyl chain containing molecular species decreased. For example, for PC the molecular species 36:6 (18:3_18:3) and 36:5 (18:3_18:2) decreased by 24% and 31%, while for PE the molecular species 36:6 and 36:5 declined by 38% and 37%, respectively, due to salt stress. On the other hand, the 18:3 acyl chain containing molecular species of TAGs increased substantially. For example, 54:8 (18:3_18:3_18:2), 54:7 (18:3_18:3_18:1), and 54:6 (18:3_18:2_18:1) increased by 220%, 198%, and 175%, respectively. These results suggest the sequestration of 18:3 acyl chains from the phospholipids into TAGs to reduce the unsaturation of the membrane phospholipids. Similar results in 18:3 acyl chain sequestration by TAGs were also reported by Spivey et al. [67] for heat stress tolerance in peanut.

MGDG, the non-bilayer forming lipid is present in plants in higher proportions and helps stabilize the major light harvesting complex (LHCII), while the bilayer forming DGDG contributes to the stacking of thylakoid membranes to form grana [93]. A stable ratio of MGDG:DGDG in the thylakoid membrane is critical for the stability and functioning of photosynthetic membranes [73]. In the present study, both MGDG and DGDG increased due to salt stress, but their ratio was stable.

PC, PE and PG have been proposed to provide a barrier for hydrophilic molecules and ions and to regulate membrane stability through their hydrophobic core [94]. An increased ratio of PC to PE suggests an elevation of membrane permeability during salt stress since PC prefers to form a stable bilayer structure, while PE forms a less stable, non-lamellar and hexagonal phase II bilayer structure [19]. In the present study, the PC to PE ratio increased for seven of the eight genotypes (Fig. 7). Although the increase was significant only for two of the salt-tolerant genotypes, a significant positive correlation (*r* = 0.776*) was observed between changes in PC:PE ratio and changes in FW due to salt treatment indicating that PC:PE ratio of membrane lipids has a role in salt tolerance in lettuce. In alfalfa, PC:PE ratio under salt stress gradually increased with longer salt stress treatment [19].

Changes in FW had a significant negative correlation with changes in PG content. Conversely, changes in FW showed significant positive correlations with the changes in content of the lipids PC, PA, TAG 16:0, TAG 18:2, TAG 18:1, and TAG 18:0. These correlations indicated that these specific lipids play an important role in salt tolerance in lettuce (Table 4). These findings were confirmed by regression analysis with changes in lipid as the predictor variable and the changes in FW as the response variable (Table 5). Results indicated that changes in FW could be predicted based on changes in lipids, particularly PG, TAG 16:0, and TAG 18:0 with corresponding *R*^2^ values of 71%, 78%, and 90% (Table 5).

Changes in DW due to salt stress also had significant positive correlations with changes in PC and TAG 18:0. Changes in the content of PS and TAG in sweet potato leaves were found to be positively associated with salt tolerance in earlier studies [59]. Significant correlations between changes in FW and changes in membrane lipid unsaturation level in the present study also indicated that the unsaturation of the lipids PG, PC, PA, TAG 16:0, TAG 18:2, TAG 18:1, and TAG 18:0 were associated with salt tolerance in lettuce. A significant positive correlation (*r* = 0.933**) between changes in the galactolipids MGDG and DGDG indicated possible coordinated metabolism of these two lipids, which can be explained by the fact that MGDG is a substrate for DGDG synthesis. Strong positive correlations among TAG 18:3, TAG 18:2, and TAG 18:1 were also observed in this study. These strong correlations could indicate coordinated metabolisms of these lipids or because the genes for these TAGs are regulated by the common transcription factor, WRINKLED1 [67, 95].

The prospects of using the lipidomics knowledge gained from this study for genetic/breeding improvement of lettuce with improved salt tolerance were explored. Study of correlations between changes/losses in FW due to salt treatment and lipid content under control condition showed that both MGDG and DGDG had significant negative correlations with FW indicating that the content of these two galactolipids were associated with losses in FW. The negative correlation indicated that the genotypes with lower MGDG and DGDG under control condition had better salt tolerance (Table S19). Both MGDG and DGDG also had significant negative correlations with most of the phospholipids, indicating that genotypes with higher amount of MGDG and DGDG had lower amount of those phospholipids (Table S19). Possible reason for this relationship can be the glycerolipid biosynthesis via the eukaryotic pathway in this 18:3 plant, where the phospholipids breakdown occurs to generate galactolipids. As the phospholipids outside of plastid also have important roles in maintaining membrane fluidity and functionality, and MGDG and DGDG have role in maintaining functionality of the photosynthetic membranes, a balance between the galactolipids and phospholipids is necessary under salt stress. Significant positive correlation between MGDG in control and MGDG in salt stress condition was observed. Similar correlation between DGDG in control and DGDG under salt stress was also observed. This indicated that lettuce genotypes with lower MGDG and DGDG content under control condition had ability to keep lower level of these two lipids under salt stress. It is possible that genotypes with lower MGDG and DGDG content that did not lead to substantial phospholipids reduction gave better salt tolerance. The relationships between changes in FW due to salt stress and the MGDG and DGDG content under control condition were also confirmed by regression analysis. The *R*^2^ values indicated that 74% and 70% variations in losses in FW were explained by MGDG and DGDG, respectively. Using this data, we developed regression models which could be used to reliably predict FW losses by the MGDG and DGDG content in the control plants (Table 6). Thus, MGDG and DGDG could possibly be used as biomarkers in selection and breeding improvement of lettuce for salt tolerance.

ANOVA for lipid content under control condition indicated presence of substantial genetic variations among the eight lettuce genotypes for most of the lipid classes studied (Tables S21 and S22). Thus, opportunities exist to breed salt tolerant lettuce cultivars by modulating desired lipid levels through genetic manipulation. Other lipidomics knowledge gained from this study could also be used, for example, manipulating the TAG biosynthesis pathway genes since higher TAGs contributed to better salt tolerance in the present study.

## Conclusions

This study demonstrates that membrane lipid remodeling is a key component of lettuce adaptation to salt stress. Salt-tolerant and salt-sensitive genotypes exhibited distinct changes in lipid content and unsaturation levels, indicating that these alterations are closely linked to salt-tolerance mechanisms. Correlation and regression analyses identified several membrane lipid classes that were significantly associated with fresh weight loss under salt stress. Using lipid profiles from control plants, regression models based on MGDG and DGDG content were developed to predict fresh weight reduction in response to salinity. These findings suggest that MGDG and DGDG are promising biomarkers for identifying and selecting salt tolerant lettuce genotypes.

## Supplementary Information

Supplementary Material 1: Table S1. The instrument parameters and the internal standards used for polar lipid analyses. Table S2. The instrument parameters and the internal standards used for the neutral lipid (TAGs) analyses. Table S3. FW, DW and Chlorophyll index raw data from three replications of eight lettuce genotypes. Table S4. List of nine fatty acids analyzed in the eight lettuce genotypes. Table S5. ANOVA for nine fatty acids involving eight lettuce genotypes and two treatments. Table S6. Effect of salt stress on nine fatty acids in eight lettuce genotypes. Table S7. Correlation among changes under salt stress of fatty acids, FW, DW and chlorophyll index. Table S8. Lipid content (normalized signal per mg sample dry weight) for 295 lipid species from 15 major lipid classes in eight lettuce genotypes. Table S9. Lipid content (Mol%) for 295 lipid species from 15 lipid classes in eight lettuce genotypes. Table S10. ANOVA of lipid content for 15 lipid classes involving eight lettuce genotypes and two treatments. Table S11. ANOVA of lipid unsaturation index for 15 lipid classes involving eight lettuce genotypes and two treatments. Table S12. ANOVA for DGDG:MGDG and PC:PE ratios involving eight lettuce genotypes and two treatments. Table S13. Mean lipid content of 15 lipid classes for eight lettuce genotypes under control and salt. Table S14. ANOVA for 130 molecular species of 15 lipid classes. Table S15. Mean lipid content of the 130 molecular species of 15 lipid classes. Table S16. Percentage change in each of the 130 lipid molecular species from 15 lipid classes in eight lettuce genotypes. Table S17. Correlation among lipid unsaturation level, FW, DW and chlorophyll index. Table S18. Correlation between changes in lipid content and changes in fatty acids content under salt stress. Table S19. Correlation between lipid content under control and FW, DW and chlorophyll index changes under salt stress. Table S20. Correlations between lipid content under control and lipid content under salt stress. Table S21. ANOVA for 15 lipid classes involving eight lettuce genotypes under control condition. Table S22. Means of lipid content for 15 lipid classes for eight lettuce genotypes under control condition.

## Data Availability

All data generated or analyzed during this study are included in this published article and its supplementary information files.

## Abbreviations

TAG: Triacylglycerol
DAG: Diacylglycerol
MGDG: Monogalactosyldiacylglycerol
DGDG: Digalactosyldiacylglycerol
SQDG: Sulfoquinovosyldiacylglycero
PA: Phosphatidic acid
PC: Phosphatidylcholine
PE: Phosphatidylethanolamine
PG: Phosphatidylglycerol
PI: Phosphatidylinositol
PS: Phosphatidylserine
LysoPC: Lysophosphatidylcholine
LysoPE: Lysophosphatidylethanolamine
FW: Fresh weight
DW: Dry weight
ER: Endoplasmic reticulum
LD: Lipid droplet
ANOVA: Analysis of variance
LSD: Least significant difference
CV: Coefficient of variation
LAU: Laura
EAB: Early Bird
MOR: Morgana
MAY: Mayfair
ERU: Eruption
PIC: Parris Island Cos
P17: PI 171676a
SHI: Shining Star
HMBS: Hoagland modified basal salt
EC: Electrical conductivity
KLRC: Kansas lipidomic research center
